# Removal of perfluoroalkyl acids and common drinking water contaminants by weak-base anion exchange resins: Impacts of solution pH and resin properties

**DOI:** 10.1016/j.wroa.2022.100159

**Published:** 2022-11-02

**Authors:** Christian Kassar, Cole Graham, Treavor H. Boyer

**Affiliations:** School of Sustainable Engineering and the Built Environment (SSEBE), Arizona State University, PO Box 873005, Tempe, AZ 85287-3005, USA

**Keywords:** Ion exchange, Natural organic matter (NOM), Per- and polyfluoroalkyl substances (PFAS), Polyacrylic, Polystyrene, Tertiary amine

## Abstract

The underlying chemistry of weak-base (WB) anion exchange resins (AERs) for contaminant removal from water is not well documented in the literature. To address this, batch adsorption experiments were conducted at pH 4, 7, and 10 using two representative WB-AERs (polyacrylic IRA67 and polystyrene IRA96) and two representative strong-base (SB) AERs (polyacrylic IRA458 and polystyrene A520E), of differing polymer composition, for the removal of nitrate, sulfate, 3-phenylpropionic acid (3-PPA) as surrogate for natural organic matter, and six perfluoroalkyl acids (PFAAs). Under acidic (pH 4) and neutral (pH 7) conditions, the selectivity of AERs for each contaminant was predominantly influenced by polymer composition followed by the size of the resin functional group. This result reflected the WB-AERs being fully protonated and functioning identical to SB-AERs. Isotherm model parameters revealed WB-AER had higher capacity than SB-AER with analogous polymer composition and porosity regardless of resin selectivity for each contaminant. Under basic conditions (≥ pH 10), contaminant removal by WB-AERs declined due to deprotonation of the tertiary amine functional groups. Removal of PFAAs by the more hydrophobic polystyrene WB-AER (IRA96) remained approximately constant with changing pH, which was possibly due to electrostatic interactions with remaining protonated amine functional groups on the resin.

## Highlights

• Weak-base (WB) and strong-base (SB) anion exchange resins (AERs) studied for pH.
• At acidic and neutral pH, similar contaminant removal by WB and SB AERs.
• Contaminant selectivity of WB AER due to polymer composition.
• Electrostatic interactions are diminished for WB AER at alkaline pH.
• Aqueous alkaline solution has great potential for regeneration of WB AER.

## 1 Introduction

Anion exchange resins are commonly used in water treatment due to facile operability, cost-effectiveness, and selectivity of specific resin structures for a range of contaminants. While the effectiveness of strong-base (SB) anion exchange resins (AERs) is well established (Dixit et al., 2021; Hu et al., 2016; Levchuk et al., 2018), the chemistry of weak-base (WB) AERs is understudied despite their benefits including strong buffering capability (Hinrichs and Snoeyink, 1976; Zhang et al., 2014), ease of regeneration (Ateia et al., 2019a), and high capacity (Boyer et al., 2021b; Höll and Kirch, 1978). WB-AERs are also less prone to organic fouling (Dixit et al., 2021), present greater chemical stability in oxidative environments, and have higher thermal resistance (Harland, 1994) than SB analogs, all of which increase resin longevity and decrease operational costs.

The main difference between SB- and WB-AERs is the basicity of their alkylamine functional groups, where WB-AERs are nonionic (tertiary amine) typically above pH ∼10 but function as SB-AER otherwise due to protonation of the resin functional group (quaternary ammonium) (Clifford and Weber, 1983). SB-AERs have been extensively studied for the removal of inorganic anions (e.g., chloride, sulfate, nitrate, perchlorate, arsenic, uranium) (Dron and Dodi, 2011a; Gu et al., 2005; Hekmatzadeh et al., 2012; Hu et al., 2016; Song et al., 2012), dissolved organic carbon (DOC) (Edgar and Boyer, 2021; Graf et al., 2014; Ness and Boyer, 2017), and hydrophobic ionizable organic compounds (Kanazawa et al., 2004; Li and SenGupta, 1998; 2004; Rahmani and Mohseni, 2017; Subramonian and Clifford, 1988), including trace emerging contaminants such as pharmaceuticals (Gustafson and Lirio, 1968; Landry and Boyer, 2013; Landry et al., 2015) and per- and polyfluoroalkyl substances (PFAS) (Boyer et al., 2021b). Significantly less research has been conducted on WB-AER, resulting in a lack of understanding regarding resin–solute–solution interactions.

While the selectivity and removal efficiency of SB-AER is generally influenced by the mobile counterion form (Laura del Moral et al., 2020) and resin properties such as polymer composition (Li and SenGupta, 1998) and functional group (Samatya et al., 2006), WB-AER exhibits additional and more complex behaviors in the absence of counterions (i.e., free-base) and across a wide pH range (Helfferich, 1995). The SB-AER-exclusive quaternary ammonium moiety is always protonated at practical pH (i.e., < 13) (Clifford and Weber, 1983; Howe et al., 2012), whereas WB-AER contains primary, secondary, and/or tertiary amines, which become deprotonated at pH greater than their acid dissociation constant (pK_a_) values losing much of their ion-exchange capacity (Clifford and Weber, 1983; Helfferich, 1995; Miyazaki and Nakai, 2011). The pH-dependent nature of WB-AER imparts promising strategies for the desorption of hydrophobic contaminants using aqueous-only alkaline solutions (e.g., NaOH, NH_4_OH, KOH) with low ecotoxicological risk (Hinrichs and Snoeyink, 1976) compared to conventional chloride salt and organic cosolvent (e.g., methanol, ethanol) solutions for SB-AER (Dixit et al., 2021; Gagliano et al., 2020), which require proper management and disposal (Boyer et al., 2021a).

Perfluoroalkyl acids (PFAAs) are a subgroup of PFAS that are amenable to removal by WB-AER given their low pK_a_ (< 1.0) and hydrophobic character (Park et al., 2020a). The human health concerns posed by PFAS are due to extensive use in industrial applications (Buck et al., 2011), exceptional resistance to biotic (Pontius, 2019) and abiotic (Tow et al., 2021) degradation, and high potential for toxicity in mammals (Abunada et al., 2020; Domingo and Nadal, 2019). Previous research evaluating the effect of resin basicity on PFAA adsorption highlighted the decreasing performance of WB-AER with increasing solution pH (Gao et al., 2017; Wang et al., 2019), which has overshadowed their treatment effectiveness and limited their use. For instance, WB-AER IRA67 exhibited up two orders of magnitude higher adsorption capacity for PFOS (Deng et al., 2010) and 9 times greater loading of PFOA than other commercial adsorbents (Du et al., 2015).

Although WB-AERs have been examined in some literature, key gaps in knowledge remain. Polyacrylic resin has been the focus of recent work investigating the pH-dependence of WB-AER for the removal of hydrophobic contaminants (Deng et al., 2010; Du et al., 2015; Shuang et al., 2012; Wang et al., 2019), where adsorption for polyacrylic AER is accompanied by a stoichiometric release of the mobile counterion (e.g., chloride) that indicates removal due to electrostatic interactions (Dixit et al., 2019, 2020; Rahmani and Mohseni, 2017). However, the influence of pH and the use of polystyrene WB-AER is understudied, despite the high uptake for hydrophobic contaminants observed by polystyrene resins even under highly alkaline conditions (Wawrzkiewicz and Hubicki, 2011; Zhang et al., 2014) that has been attributed to non-electrostatic adsorption such as hydrophobic interactions and van der Waals forces (Landry et al., 2015). When chloride was the mobile counterion, polyacrylic WB-AER had three times higher adsorption capacity for lactic acid compared to the resin in free-base form, whereas polystyrene WB-AER had less than two times, similarly explained by the absence of electrostatic interactions (Moldes et al., 2003). An enhanced understanding of the influence of electrostatic and non-electrostatic interactions between WB-AER and contaminants with different properties is needed to devise a framework for effective treatment and regeneration. The adsorption mechanism, selectivity, and capacity of AERs are generally deduced from isotherm models. However, using linear regression to determine model parameters may lead to misinformed conclusions (Allen et al., 2003; Bolster and Hornberger, 2007; Dron and Dodi, 2011a; Foo and Hameed, 2010; Haupert et al., 2021; Tran et al., 2017b).

The overall goal of this work was to gain new insights on the chemistry of WB-AER for contaminant removal from water. The specific objectives of this research were to (1) investigate the impact of solution pH and resin properties such as resin basicity, polymer structure, and functional group, on the removal efficiency; (2) evaluate the influence of PFAAs properties, in term of perfluorocarbon tail lengths and head group, on the selectivity sequence for each AER; (3) discuss the underlying mechanisms and effectiveness of chloride-form WB-AER for various water pollutants; and (4) assess the reliability of different isotherm modeling techniques for single-solute ion-exchange systems. The improved understanding presented in this research benefits municipal and industrial implementation of WB-AER for various contaminants encountered in water.

## 2 Materials and methods

### 2.1 Anion exchange resins

Four AERs were used in this research with their properties listed in Table 1. Weak-base Amberlite IRA67 (WB/PA/G/dimethyl) and weak-base Amberlite IRA96 (WB/PS/MP/dimethyl) resins were obtained in free-base form. Strong-base Amberlite IRA458 (SB/PA/G/trimethyl) and Purolite A520E (SB/PS/MP/triethyl) were obtained in chloride-form. For consistency, all AERs were pretreated with 10× more Cl^−^ than the exchange capacity of the resin then repeatedly washed with deionized (DI) water. A sodium chloride (NaCl)/hydrochloric acid (HCl) solution was used to treat the SB- and WB-AERs to assist with the protonation of tertiary amine functional groups. Resin density was determined by measuring 20 mL of wet settled resin in a graduated cylinder. Dry density was calculated as the ratio of dry mass to wet volume after oven-drying the resins at 55 ℃ for 24 h.

**Table 1:** Characteristics of anion exchange resins used in this research.

Resin	Basicity	Polymer composition/Pore structure	Functional group	Exchange capacity (eq/L)^a^	Exchange capacity (meq/g)^b^	pK_a_	Water Content (%) at pH 7
IRA67	WB	PA/G	Tertiary amine: Dimethyl^c^	1.6	4.56	9.0^f^	56–64^h^
IRA96	WB	PS/MP	Tertiary amine: Dimethyl^c^	1.25	3.29	6.4^f^	57–63^h^
IRA458	SB	PA/G	Type I: Trimethyl^d^	1.25	4.10	>13 g	57–64^i^
A520E	SB	PS/MP	Type I: Triethyl^e^	0.9	2.44	>13 g	50–56^i^
Strong-Base (SB), Weak-Base (WB), Polyacrylic (PA), Polystyrene (PS), Gel (G), Macroporous (MP).							

^a^Data obtained from the manufacturer.

^b^Determined experimentally.

^c^R-(CH_3_)_2_HN^+^ (protonated form) or R-(CH_3_)_2_N (free-base form).

^d^R-(CH_3_)_3_N^+^.

^e^R-(CH_2_CH_3_)_3_N^+^.

^f^(Miyazaki and Nakai, 2011).

^g^(Clifford and Weber, 1983).

^h^Manufacturer data for resin in free-base form.

^i^Manufacturer data for resin in chloride form.

### 2.2 Chemical analytes

Synthetic solutions in both single- and multi-solute experiments were prepared by dissolving approx. 2.14 meq/L of total analytes in DI water (resistivity >18.2 MΩ-cm). The solution pH was adjusted using 1M HCl and 1M NaOH solutions. The single-solute experiments included sodium nitrate (NaNO_3_, CAS# 7631-99-4, Fisher Scientific), 3-phenylpropionic acid (C_8_H_9_COOH, CAS# 501-52-0, Alfa Aesar), and sodium sulfate (Na_2_SO_4_, CAS# 7757-82-6, Fisher Scientific). 3-phenylpropionic acid (3-PPA) is a small organic molecule that can form from the degradation of high molecular weight compounds such as natural organic matter (NOM) (Shuang et al., 2015). In this work, 3-PPA was used as surrogate for NOM due to structural similarities. Specifically, 3-PPA contains the core group of NOM, namely a hydrophobic aromatic carbon moiety and a hydrophilic carboxylic acid functional group (Chin et al., 1994). The multi-solute experiment consisted of six PFAAs with varying perfluorocarbon tail lengths and head groups, each at an initial concentration of 80 μg/L to reflect typical amounts (i.e., tens of μg/L) in groundwater amenable to source zone contamination (Kärrman et al., 2011; Xu et al., 2021) from fire-fighting activities (McGuire et al., 2014; Yao et al., 2014) and fluorochemical facilities (Lin et al., 2009). To provide meaningful comparison between the equilibrium tests, sodium bicarbonate (NaHCO_3_, CAS# 1066–33–7, Sigma Aldrich) was added to the mixture to a final equivalent concentration of analytes $\left(C_{\sum X^{-}}\right)$ of 2.14 meq/L (Millar et al., 2015). The PFAAs in the order of decreasing number of carbons (C#) were perfluorooctane sulfonate (C8, PFOS, aqueous, CAS# 1763-23-1), perfluorooctanoic acid (C8, PFOA, solid, CAS# 335-67-1), perfluorohexane sulfonate (C6, PFHxS, sodium salt, CAS# 3871-99-6), perfluorohexanoic acid (C6, PFHxA, aqueous, CAS# 307-24-4), perfluorobutane sulfonate (C4, PFBS, sodium salt, CAS# 29420-49-3), and perfluorobutanoic acid (C4, PFBA, aqueous, CAS# 375-22-4) and were purchased from Sigma-Aldrich at ACS grade. The stock solution was prepared as a mixture of the six PFAAs at 32 mg/L each in DI water and was further sonicated to achieve full dissolution.

### 2.3 Batch adsorption experiments

Adsorption experiments were conducted in a benchtop orbital shaker (Thermo Scientific™ MaxQ™) for 24 h (at ambient laboratory temperature) with dry resins in 125 mL amber glass bottles containing 100 mL of synthetic contaminant solution. The percent resin dose was defined as the ratio of resin dose (meq/L) to initial total concentration of analytes (meq/L). Five resin doses (25%, 50%, 100%, 150% and 300%) were selected for this study. Batch equilibrium tests were carried out at constant concentration of 2.14 meq/L by varying the resin mass, which showed to be a better representation of the ion-exchange process than varying the initial concentration of analytes at same resin mass (Millar et al., 2015). The contaminant solutions were prepared at three different solution pH (4, 7, and 10). To investigate the influence of alkaline conditions on WB-AER removal efficiency, WB resins were contacted with a highly basic solution (pH ∼11) for 24 h then carefully decanted. The test water at pH 11.4 was subsequently added to the resin and equilibrated for 24 h. This method was adopted for WB-AER to compensate for the deprotonation of amine groups, which constantly decreases the pH in unbuffered test water (Gustafson et al., 1970). For simplicity, the two methods used to equilibrate WB- and SB-AERs in basic conditions are referred to as pH 10 in this paper. All samples were tested in triplicate and the pH was measured before and after adsorption experiments. Control samples with no resin showed negligible loss of contaminant due to adsorption to glass walls.

### 2.4 Analytical methods

Concentrations of Cl^−^, nitrate (NO_3_^−^), and sulfate (SO_4_^2−^) anions, and sodium (Na^+^) cations were measured using ion chromatography (IC) (Dionex ICS 5000+, Sunnyvale, California) as described elsewhere (Edgar and Boyer, 2021). Cl^−^ concentration was determined to calculate the separation factor of AER for binary IX systems (i.e., Cl^−^/NO_3_^−^, Cl^−^/3-PPA, Cl^−^/SO_4_^2−^). Single-analyte samples were passed through a 0.45 µm nylon membrane filter. Cellulose acetate syringe filters were used for multi-analyte samples since they exhibit the lowest PFAAs losses to the filter material in DI water matrices (Sörengård et al., 2020). All samples were stored in conical polypropylene tubes with zero head space at 5℃. DOC and dissolved inorganic carbon (DIC) were measured as surrogates for 3-phenylpropionic acid (3-PPA) and HCO_3_^−^, respectively using a Total Organic Carbon analyzer (TOC-VCH, Shimadzu, Japan). Reported results are the averages of duplicates with relative percent difference (RPD) below 10%. During each run, organic carbon standard (1000 ppm C, CAS# 1847-16, Ricca Chemical) and inorganic carbon standard (1000 ppm C, CAS# 1845-4, Ricca Chemical) were used periodically as checks (RPD <15%). Some 3-PPA samples were tested for UV absorbance at 254 nm using a UV–Visible Spectrophotometer (UV-2700, Shimadzu) and showed DOC measurements to be representative of the 3-PPA isolate. The calibration curve (R^2^ > 0.995) was made from several concentrations of 3-PPA (30, 50, 100, 200, 250, and 400 mg/L) in DI water. High-performance liquid chromatography (HPLC 1290 Infinity II, Agilent Technologies) coupled to a triple quadruple mass spectrometer (LC/MS 6490, Agilent Technologies) was employed for PFAA analysis. A 5 μL sample was injected onto a C18 analytical column (5 μm, 100×3 mm, 110 Å, Phenomenex Gemini) protected by a C18 guard column (4 × 2 mm, Phenomenex Gemini), which was replaced at intervals of 100 injections. To ensure the retention of short-chain PFAAs (i.e., PFBA, PFBS and PFHxA), Phenomenex C18 equipment were separated by two hydrophilic DIOL guard columns (6 μm, 4.6 × 12.5 mm, Agilent Technologies). A C18 delay column (5 μm, 30×3 mm, 100 Å, Luna) was used to enhance analyte separation and minimize contamination. The eluent consisted of 20 mM ammonium acetate (CAS# 631-61-8) in water (CAS# 7732-18-5), as the aqueous mobile phase, and methanol (CAS# 67-56-1), as the organic mobile phase. All eluent reagents were purchased from Fischer Scientific at Optima HPLC-grade. The mixture flow rate was maintained at 0.8 mL/min.

### 2.5 Data analysis

The equivalent concentration of each analyte in the resin phase (q_e_; meq/g) was calculated on a mass balance basis from Eq. (1):

(1) $$q_{e}=\frac{\left(C_{0}-C_{e}\right)}{m}\;v$$

where $C_{0}$ (meq/L) and $C_{e}$ (meq/L) are the initial and equilibrium aqueous concentration of the target analyte, respectively, m (g) is the dry mass of resin, and v is the volume of synthetic solution. The removal efficiency (%) at each percent resin dose was determined as the amount of contaminant removed from solution normalized by initial concentration. To account for additional chloride sites, stemming from resin pre-conditioning, the resin capacity was recalculated for all resins using 10× more NO_3_^−^ and 10× more SO_4_^2−^ than the manufacturer value. The highest exchange capacity for each AER was then used for determination of isotherm model parameters (see Electronic supplementary material). This recalculation was due to higher observed contaminant uptake and chloride release for low percent resin dose than the maximum theoretical resin capacity. Previous work reported polyacrylic SB-AER to have capacity approx. 2× the manufacturer value (Hu et al., 2016). Percent removal, q_e_, and separation factors for each percent resin dose and analyte were computed as the average of triplicate samples with error bars representing one standard deviation.

To investigate the controlling adsorption mechanism responsible for contaminant removal, the experimental q_e_ data were fit to the Langmuir, Freundlich, Dubinin-Radushkevich (DR), Dubinin-Astakhov (DA), and Redlich-Peterson (RP) isotherm models. The Langmuir isotherm model, expressed in Eq. (2), is widely used to fit ion-exchange equilibrium data due to the ease and usefulness of estimating the model parameters (Dron and Dodi, 2011b; Song et al., 2012).

(2) $$q_{e}=\frac{q_{0}K_{L}C_{e}}{1+K_{L}C_{e}}$$

where q_0_ (mmol/g) is the maximum theoretical capacity of the resin and K_L_ (L/mmol) is the Langmuir coefficient associated with ion-exchange selectivity. The fitting degree of isotherm models were evaluated based on goodness-of-fit parameters. Isotherm models and goodness-of-fit parameters are discussed in detail in supplementary material.

## 3 Results and discussion

### 3.1 Effect of solution pH on contaminant removal by WB-AER

**Fig. 1:**
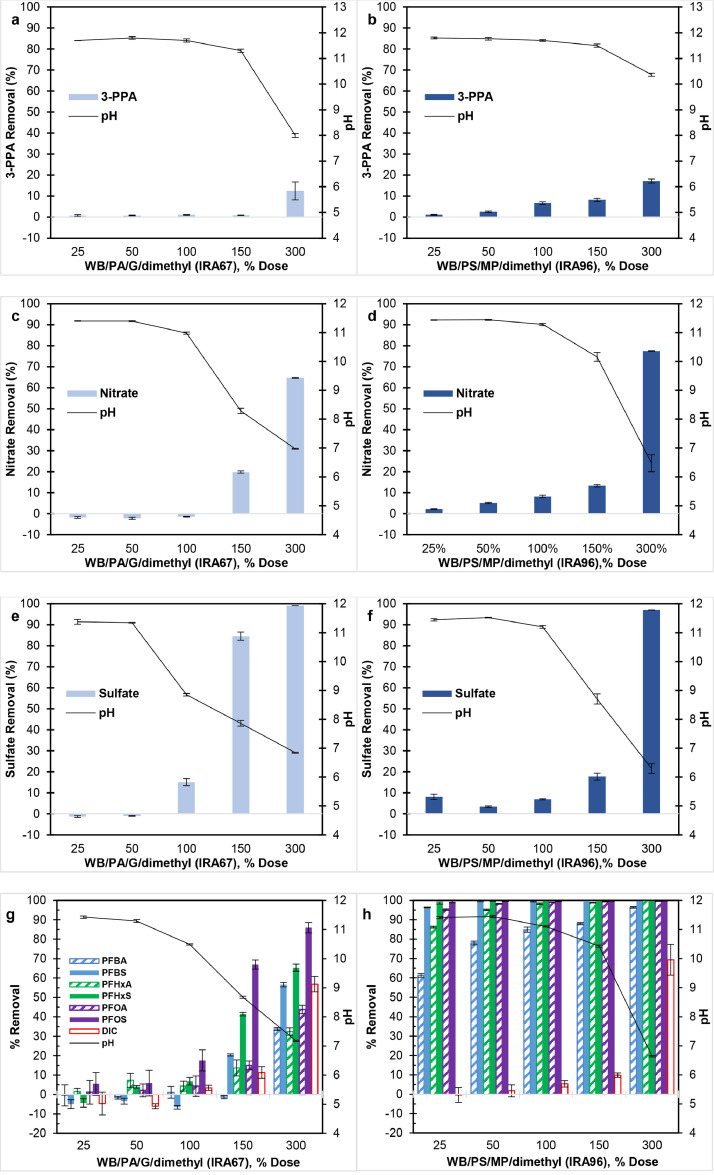
Impact of solution pH on contaminant removal by weak-base anion exchange resins for (a, b) 3-phenylpropionic acid, (c, d) nitrate, and (e, f) sulfate in single-solute system (C_0_ ≈ 2.14 meq/L) and (g, h) the six PFAAs in the presence of sodium bicarbonate (C_0_ ≈ 2.14 meq/L) in multi-solute system. Initial concentration of each PFAA was C_0_ = 80 μg/L (∑PFAAs = 480 μg/L). Resins were first equilibrated for 24 h under basic conditions (pH ≈ 11) then placed in test water at: (a, b) pH 11.7, (c, d) pH 11.4, (e, f) pH 11.4, and (g,h) pH 11.4. Error bars show one standard deviation.

Fig. 1 shows the impact of solution pH on contaminant removal by WB-AER. The WB-AERs showed <10% removal of 3-PPA (Fig. 1a and b), nitrate (Fig. 1c and d), sulfate (Fig. 1e and f), and DIC (Fig. 1g and h) at solution pH >10. The pH decreased as more resin was added to the test water due to the increasing number of deprotonating WB-AER functional groups (i.e., H^+^ release). At the 300% dose, both WB/PA/G/dimethyl resin (IRA67) and WB/PS/MP/dimethyl resin (IRA96) reached removals of ≥97% sulfate, ≥60% nitrate, and ≥55% DIC at pH 6–7 and ≥10% 3-PPA at pH 8–10.4. The higher contaminant removal at lower pH and higher resin dose is consistent with increasing protonated functional groups of the WB-AER. At resin dose ≤100%, H^+^ released from the resin was neutralized by excess OH^−^ in solution, which kept the pH stable at pH ∼10 and the functional groups uncharged. At higher resin doses, H^+^ was in excess relative to OH^−^ thereby decreasing the solution pH to 6–8. The WB-AERs function as nonionic resins as pH surpasses the pK_a_ and electrostatic interactions diminish. The addition of sodium bicarbonate to the PFAA mixture contributed to 107 mg/L as CaCO_3_ of alkalinity, which is on the low end of realistic groundwater and was not sufficient to buffer the pH. The pH values measured at the 300% dose and the pK_a_ values estimated from previous studies were in closer agreement for IRA96 (pH = 6.3–6.6; pK_a_ = 6.0–6.3) than IRA67 (pH = 6.8–7.2; pK_a_ = 9.3) (Macpherson, 2009; Miyazaki and Nakai, 2011; Zhang et al., 2014).

The WB/PA/G/dimethyl resin (IRA67; Fig. 1g) followed the same trend for PFAAs as the other contaminants, while the WB/PS/MP/dimethyl resin (IRA96; Fig. 1h) was not affected by solution pH and still achieved 90% removal at pH >10. In fact, WB/PS/MP/dimethyl resin consistently showed measurable removal of all contaminants even when solution pH was much greater than the resin pK_a_. While the polystyrene composition could have allowed IRA96 to adsorb PFAAs and 3-PPA via hydrophobic interactions and/or van der Waals forces, sulfate, nitrate, and bicarbonate are only bound electrostatically to the resin, which suggested that IRA96 had a fraction of protonated functional groups with higher pK_a_. Since the initial PFAA concentration of 480 μg/L was substantially exceeded by the resin dose, electrostatic interactions with remaining charged amine groups of the IRA96 were sufficient to achieve >95% removal of all PFAAs at the 50% resin dose. In a previous study, multiple asymmetric peaks were detected over the ^31^P NMR spectral range of IRA96 indicating heterogeneity in amine functionalities of the resin structure (Miyazaki and Nakai, 2011). In another study, some WB-AERs had polyamine functional groups while commercially advertised as secondary or tertiary amine resins (Clifford and Weber, 1983). Such non-uniformity arises from the difficulties of controlling the chloromethylation step during resin synthesis (Anderson, 1964).

The results in the literature showed similar decrease in WB-AER uptake of inorganic anions (Awual et al., 2008; Kołodyńska, 2009, 2010), hydrophobic contaminants (Greluk and Hubicki, 2011; Gustafson and Lirio, 1968; Hinrichs and Snoeyink, 1976; Wawrzkiewicz, 2011; Wawrzkiewicz and Hubicki, 2011; Zhang et al., 2014), and PFAS (Deng et al., 2010; Du et al., 2015; Gao et al., 2017; Wang et al., 2019; Yang et al., 2018) with increasing solution pH. However, the results of this study clearly demonstrated the reversibility of this behavior in batch conditions, whereby a decrease in solution pH resulted in protonation of amine functional groups and increasing removal thereupon. These findings justify pre-treating WB-AERs with solutions of strong acids such as HCl as described elsewhere (Bolto et al., 2002; Moldes et al., 2003; Sengupta and Clifford, 1986) and precludes using free-base form WB-AERs in unbuffered test water with low ionic strength. WB-AER could bind electrostatically to charged contaminants only when H^+^ and a mobile counterion simultaneously occupy AER sites (Helfferich, 1995). For instance, a study showed a 10-fold decrease in the adsorption capacity for PFOS on free-base IRA67 resin compared with its chloride-form Gao et al. (2017).

Although IRA67 (WB/PA/G/dimethyl) demonstrated no adsorption capacity for contaminants at pH ∼11.5, the resin exhibited >50% removal of all PFAAs and greater removal of PFSAs than PFCAs when solution pH decreased to 7.2 (Fig. 1g). The trends for PFAA removal are consistent with stronger electrostatic interactions between more negatively charged sulfonate than carboxylate head group of PFAA (Ateia et al., 2019b) and increasing number of protonated resin functional groups at lower pH. These results indicate that WB-AER could be effectively used for PFAA treatment given that the majority of drinking water applications involve water sources having pH <9.0 (Du et al., 2014; Gagliano et al., 2020) while higher pH applications are mostly for regeneration purposes using alkaline solutions. More importantly, the low removal of PFAAs under highly basic conditions (i.e., pH 11.5) suggested no available ion-exchange sites on the resin due to complete deprotonation of amine functional groups and thus the potential for effective regeneration using dilute NaOH and/or NaOH brine solutions (Hinrichs and Snoeyink, 1976; Jackson and Bolto, 1990). Ateia et al. (2019a) used various nonionic adsorbents, such as highly porous nanostructured polymers, to highlight the benefits of deprotonation of tertiary amine functional groups in alkaline conditions. Likewise, effective PFAS desorption from IRA67 was achieved using 0.04–1% NaOH (Du et al., 2015; Gao et al., 2017; Wang et al., 2019), with an interesting result showing increasing desorption efficiency with decreasing % NaOH as 4% NaOH > 0.4% NaOH > 0.04% NaOH (Deng et al., 2010). While single-use resins have been used for the removal of PFAS at trace levels (ng/L) (Fang et al., 2021; Zaggia et al., 2016), regenerable resins have been pursued for the remediation of waters containing PFAS concentrations ranging from tens to several hundreds of μg/L (Liu and Sun, 2021; Woodard et al., 2017). The results of the current work suggest that the fraction of NaOH could be decreased to 0.015% (pH ∼11.57), thus reducing chemical requirements and thereby operating costs for drinking water applications involving high concentrations of PFAAs at μg/L levels. However, this does not pertain to IRA96 (WB/PS/MP/dimethyl), which showed high affinity for PFAAs regardless of pH. In addition, a previous study showed that the selectivity for hydroxide ions depends on the equilibrium concentration of NaOH in solution and resin properties such as porosity and polymer composition (Höll and Kirch, 1978), which could explain the different pH trends in the current study. To confirm these conjectures, studies involving continuous-flow column regeneration of WB-AER using alkaline solutions are the next logical steps in this research.

### 3.2 Effect of resin properties on individual contaminant removal

**Fig. 2:**
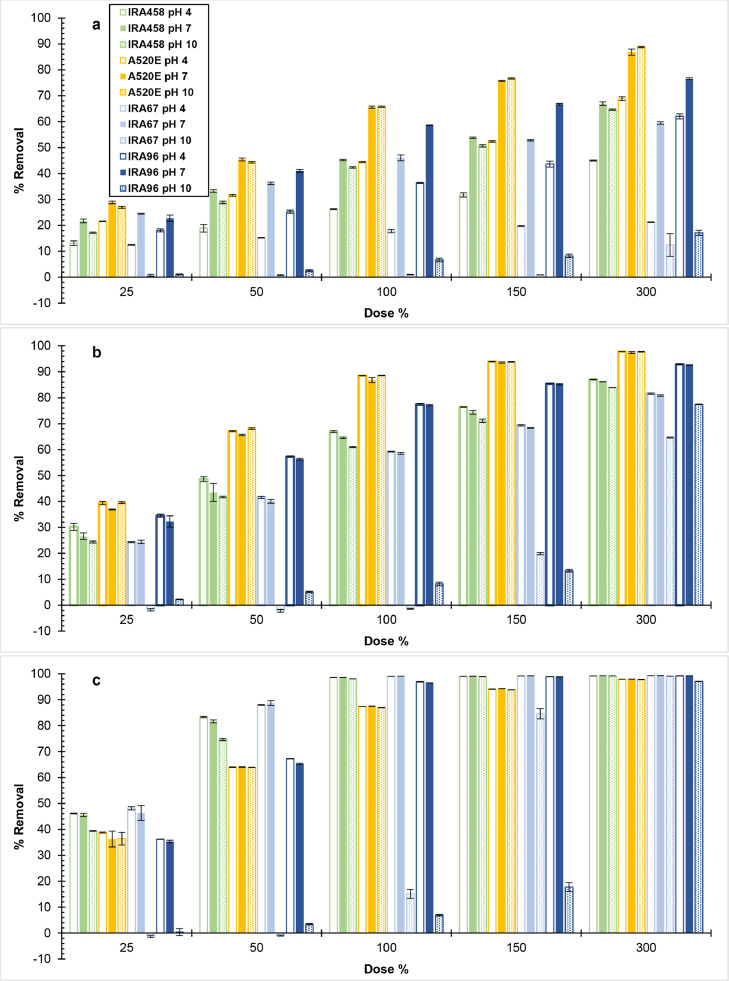
Removal of (a) 3-phenylpropionic acid, (b) nitrate, and (c) sulfate by AER at different solution pH (4, 7, and 10) in single-solute system. Initial contaminant concentration C_0_ = 2.14 meq/L. Percent resin dose (Dose %) is the ratio of theoretical resin capacity to initial contaminant concentration on equivalent basis. Bars are the mean of triplicate samples with error bars showing one standard deviation.

IRA458 (SB/PA/G/trimethyl), A520E (SB/PS/MP/triethyl), IRA67 (WB/PA/G/dimethyl), and IRA96 (WB/PS/MP/dimethyl) resins were tested to investigate the effect of resin characteristics for separate removal of contaminants. Fig. 2 shows the percentage removal of 3-phenylpropionic acid (3-PPA), nitrate, and sulfate at pH 4, 7, and 10 as a function of resin dose from 25% to 300%. At a given resin dose, nitrate and sulfate exhibited similar removal by SB-AERs regardless of solution pH (4, 7, and 10) and by WB-AERs at solution pH 4 and 7. The same removal trend in terms of solution pH held for 3-PPA except for the lower removal observed at pH 4. The WB-AERs showed no to low removal of contaminants at pH 10 (see Section 3.1). These results indicated the tertiary amine functional groups of the WB-AERs behaved similarly as the quaternary ammonium functional groups of the SB-AERs at pH ≤ 7. Previous findings also corroborated minor change in adsorption capacity of AERs with quaternary ammonium and tertiary amine functional groups over solution pH 3–8 (Deng et al., 2010; Gao et al., 2017; Greluk and Hubicki, 2011), which is of importance since most water bodies are at pH < 8. The reason for the lower removal of 3-PPA under acidic than neutral conditions (20% removal at the 100% dose) was due to a portion of the contaminant being in the conjugate base form (pK_a_ = 4.66 at 25 °C) (Kortüm et al., 1960), which is consistent with weaker electrostatic interactions between the carboxylic acid moiety of 3-PPA and the resin functional group. Hence, removal of weakly acidic contaminants by WB-AERs must consider the acid-base properties of both the contaminant and resin.

The following results are specific to pH 4 and pH 7 and are referred to as nitrate, sulfate, 3-PAA at pH 7, and 3-PPA at pH 4. Similar removal was observed between IRA458 (SB/PA/G/trimethyl) and IRA67 (WB/PA/G/dimethyl) (<10% difference) under all pH conditions and resin dose except 3-PPA at pH 4. Briefly, the functional groups of the selected WB-AERs and Type I SB-AERs were dimethylamine R-(CH_3_)_2_HN^+^ (IRA67 and IRA96) and trialkylamine, with SB-AER groups further subdivided into triethyl R-(CH_2_CH_3_)_3_N^+^ (A520E) and trimethyl R-(CH_3_)_3_N^+^ (IRA458). These results show no influence of AER basicity on resin performance at pH ≤7, suggesting that tertiary amine and quaternary ammonium functional groups with same alkylamine chain length impart similar adsorption properties to the resin. The higher observed removal of 3-PPA by dimethyl compared to trimethyl-functionalized PA resin at pH 4 was due to the WB-AER release of H^+^ in unbuffered solution. Several studies compared the impact of resin basicity for the removal of charged organic dyes and showed IRA458 (SB/PA/G/trimethyl) to have half (Wawrzkiewicz, 2011), seven (Greluk and Hubicki, 2011), and ten times (Greluk and Hubicki, 2010; Wawrzkiewicz and Hubicki, 2011) more maximum capacity than IRA67 (WB/PA/G/dimethyl). Reasons for these differences were the use of non-normalized data, different resin dose, and contaminant concentration. In this work, resin dose (%) accounted for the capacity of the resin (see Section 2.3), which implied AER selectivity. A previous study supported all these statements, whereby WB/PA/dimethyl resin had higher loading capacity (meq/g) and similar selectivity (i.e., normalized loading) for various hydrophobic contaminants than SB/PA/trimethyl resin (Gustafson and Lirio, 1968).

Nitrate and 3-PPA at pH 7 and 3-PPA at pH 4 followed the same trend for all AERs (Fig. 2a, b) with the respective order of decreasing removal as SB/PS/MP/triethyl (87%, 66%, and 44%) > WB/PS/MP/dimethyl (77%, 59%, and 36%) > SB/PA/G/trimethyl (65%, 45%, and 26%) ≈ WB/PA/G/dimethyl (59%, 46%, and 18%). The order of decreasing sulfate removal (Fig. 2c) before reaching complete uptake was WB/PA/G/dimethyl (88–89%) > SB/PA/G/trimethyl (82–83%) > WB/PS/MP/dimethyl (65–67%) ≈ SB/PS/MP/triethyl (64%). As a rule, the affinity of the solute is highest for the resin with complementary polar character. The polystyrene matrix of the resin is composed of a repeating (CH_2_)-CH-(CH_2_)-benzene unit making it more hydrophobic than polyacrylic resin with benzene rings substituted by carbonyl groups. A520E (SB/PS/MP/triethyl) is slightly more hydrophobic than IRA96 (WB/PS/MP/dimethyl) due to the longer alkylamine chains of ethyl than methyl functional groups (Helfferich, 1995; Zaggia et al., 2016). Sulfate is ∼4 times more hydrated than nitrate (Marcus, 1991), thus immobilizing water molecules within the hydration shell more effectively. However, hydrogen bonds with water molecules are less likely to form in the case of 3-PPA given the nonpolar phenyl moiety attached to an aliphatic chain (Li and SenGupta, 1998). Hence, 3-PPA and nitrate had higher affinity to polystyrene resin than polyacrylic resin, and to A520E (PS/triethyl) than IRA96 (PS/dimethyl), whereas sulfate adsorption was more favorable by polyacrylic resins.

At the stoichiometric resin dose (i.e., 100%), all AERs exhibited >96% sulfate removal apart from A520E (SB/PS/MP/triethyl) with ∼88% removal implying the preference of resins with closely spaced alkylamine groups for divalent over monovalent ions (Clifford and Weber, 1983; Subramonian and Clifford, 1988). The trends comparing alkylamine chain length and polymer composition of the resin are validated by ΔG^0^ values for the adsorption of hydrophobic contaminants (Landry and Boyer, 2013; Landry et al., 2015; Li and SenGupta, 2004) and inorganic anions (Hu et al., 2016) on AERs with structures similar to the ones tested in this study. The length of the resin functional group could also affect adsorption kinetics (Gu et al., 2000), but it was not included in this research.

The water content of the free-base WB-AERs and chloride-form SB-AERs considered in this study are similar ranging 50–64% (Table 1). However, the degree of resin swelling, as expressed by its water content, increases by up to 20% upon conversion of free-base WB-AERs to the chloride-form and decreases equally in solutions of high ionic strength (e.g., extreme pH conditions) (Harland, 1994), all of which are important considerations for the removal of high molecular weight organic compounds such as NOM (Bolto et al., 2002; Shuang et al., 2015) but are less relevant for smaller contaminants (e.g., 3-PPA, PFAA) (Laura del Moral et al., 2020). Additionally, previous studies have shown AER porosity to have no influence on contaminant removal in batch equilibrium tests. This is because macroporous and gel resins vary in pore size distribution (50–100 nm vs. <2 nm) (Dudley, 2012) and surface morphology, which affect adsorption kinetics (Li and SenGupta, 2000) and not adsorption equilibrium. The results indicated that the hydrophobicity (polymer composition and functional group length) and spacing between functional groups of the resin were the key properties influencing contaminant selectivity as opposed to resin basicity (i.e., tertiary amine vs. quaternary ammonium), which showed no measurable impact at pH ≤7.

### 3.3 Competitive removal of six PFAAs

**Fig. 3:**
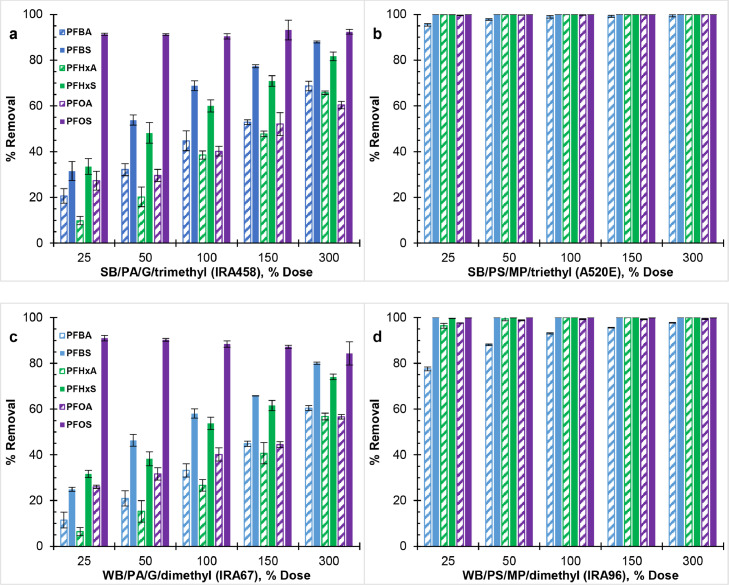
Effect of resin polymer composition on perfluoroalkyl acids (PFAAs) removal by (a,c) polyacrylic, and (b,d) polystyrene AERs at pH 4. Initial concentration of each PFAAs was C_0_ = 80 μg/L (∑PFAAs = 480 μg/L) Bicarbonate pK_a_ = 6.3 was mostly in the neutral CO_2 (aq)_/H_2_CO_3_ form at pH 4.

**Fig. 4:**
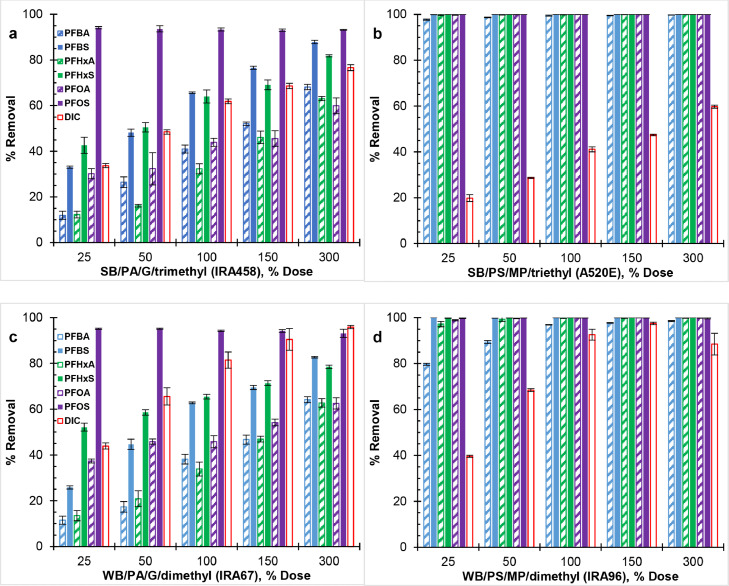
Effect of resin polymer composition on perfluoroalkyl acids (PFAAs) removal by (a,c) polyacrylic, and (b,d) polystyrene AERs in the presence of sodium bicarbonate (C_0_ ≈ 2.14 meq/L) at pH 7. Initial concentration of each PFAAs was C_0_ = 80 μg/L (∑PFAAs = 480 μg/L).

Figs. 3, 4, and S1 show the effect of resin properties on the co-removal of PFAAs at different solution pH. WB-AERs and SB-AERs with analogous properties (i.e., IRA67/IRA458 and IRA96/A520E) had same removal results for PFAAs at pH ≤7, which indicates no impact of resin basicity on resin hydrophobicity and thereby PFAAs selectivity. Non-electrostatic interactions play important role in resin selectivity and become more pronounced with increasing hydrophobicity of the resin (Boyer et al., 2021b). However, due to the more hydrophobic character of ethyl than methyl alkylamine functional group, A520E (SB/PS/MP/triethyl) was expected to remove PFAAs to a greater extent than IRA96 (WB/PS/MP/dimethyl) (Conte et al., 2015; Zaggia et al., 2016). Given the significant removal of PFAAs (∼100%) by polystyrene AERs at all resin doses, the impact of functional group length of the resin on PFAA selectivity was barely measurable. The results on resin hydrophobicity are supported by a previous study showing the order of decreasing normalized loading (i.e., selectivity) for PFOS as triethylammonium > trimethylammonium ≈ dimethylamine (Schuricht et al., 2017).

Figs. 3 and 4 show equal removal at pH 4 and pH 7 for all AERs. IRA96 (WB/PS/MP/dimethyl) and A520E (SB/PS/MP/triethyl) resins maintained their performance with respect to PFAAs removal over the range of pH but decreased by up to 90% for IRA67 (WB/PA/G/dimethyl) and by 5–50% for IRA458 (SB/PA/G/trimethyl) at pH 10 (see Fig. S1). Because the pK_a_ of selected PFAAs spans from −3.33 to 1.07 (Maimaiti et al., 2018; Park et al., 2020a), any effect on removal efficiency was attributed to either the protonating state of amine functional group of the resin or the speciation of DIC (i.e., carbonic acid/bicarbonate/carbonate). Inorganic carbon species were mostly in the neutral CO_2 (aq)_/H_2_CO_3_ form at pH 4 (pKa = 6.3) and partly as carbonate ions (CO_3_^2−^) at pH 10 (pKa = 10.3). This indicated no impact of HCO_3_^−^ on PFAAs removal for all AERs (i.e., pH 4 vs. pH 7), whereas the lower removal by the polyacrylic IRA458 could be due to competing CO_3_^2−^ and OH^−^ at low PFAAs concentration. Since IRA458 (SB/PA/G/trimethyl) showed similar DIC removal on molar basis between pH 7 and pH 10, more ion exchange sites were occupied by divalent CO_3_^2−^ decreasing the ones that were available for PFAAs. This is supported in previous research, where PFOA removal was reduced by less than 8% but nearly 27% after separate addition of 1 meq/L HCO_3_^−^ and CO_3_^2−^ (Yang et al., 2018). Other studies that have investigated the efficiency of SB-AER under different pH conditions are in major disagreement (Deng et al., 2010; Dixit et al., 2019; Maimaiti et al., 2018; Wang et al., 2019; Yu et al., 2009) with Dixit et al. (2019), Wang et al. (2019), and Yu et al. (2009) showing decreasing PFAS removal with increasing pH but with no clear consensus.

At pH 4 and pH 7, all AERs achieved >85% PFOS removal regardless of resin dose (i.e., 25 to 300%) or resin properties. Across all resin doses, PFOS removal by polyacrylic AERs was consistent and ranged from 86 to 95%, whereas polystyrene AERs exhibited complete removal (∼100%). The high value of the pH-corrected octanol-water partition coefficient (log D_ow_) for PFOS (Table S1) is indicative of its substantially more hydrophobic and less water soluble character than remaining PFAAs (i.e., two orders of magnitude) (Park et al., 2020a; Zeng et al., 2020). PFOS partitions even into relatively polar resin (e.g., polyacrylic) in response to a driving force while the polystyrene resin has inherent affinity for hydrophobic compounds (Dietz et al., 2021). PFAAs become more hydrophobic (i.e., less polarizable) as the number of electron-withdrawing C-F_n_ group increases given the highly nonpolarizable character of fluorine atoms (i.e., high electron binding energy) (Liu and Sun, 2021; Moody and Field, 2000). Similarly, PFSAs are more hydrophobic than PFCAs with same number of carbons due to the more polarizable carboxylate (–COO^−^) than sulfonate (–SO_3_^−^) head groups and the additional C-F_n_ group residing on the carbon tail of PFSAs (Du et al., 2014; Park et al., 2020b). The differences across PFAA structures in terms of number of C-F_n_ and head group are in line with the order of decreasing log D_ow_ listed in Table S1 as PFOS (8 C-F_n_; –SO_3_^−^) > PFHxS (6 C-F_n_; –SO_3_^−^) ≈ PFOA (7 C-F_n_; –COO^−^) > PFBS (4 C-F_n_; –SO_3_^−^) ≈ PFHxA (5 C-F_n_; –COO^−^) > PFBA (3 C-F_n_; –COO^−^). Nonetheless, the selectivity of polyacrylic AERs was not completely consistent with the perfluorinated carbon tail length and thus the hydrophobic character of PFCAs and PFSAs. Because all PFAAs had same initial mass concentration (80 μg/L), low molecular weight PFAAs were present at higher equivalent concentration than larger compounds (e.g., PFBA-to-PFOA ratio of ∼2) (see Table S1), which made exact comparison in terms of carbon chain length difficult. Although previous studies have shown that high molecular weight PFAAs could block resin pores limiting the intraparticle diffusion of smaller counterparts at low resin-to-PFAA dose (e.g., 0.2 meq resin/meq PFAA) (Maimaiti et al., 2018; Schuricht et al., 2017), the number of ion-exchange sites in this study exceeded 370× the total concentration of PFAA at the smallest resin dose (i.e., 25%), thereby providing access for all PFAAs.

Other trends include the greater removal of PFAAs by polystyrene (IRA96 and A520E) than polyacrylic (IRA67 and IRA458) resins, and of PFSAs than PFCAs by polyacrylic resins which are consistent with the nonpolar character of the polystyrene resin matrix and the stronger ionic charge of sulfonates than carboxylates head groups (Ateia et al., 2019b; Park et al., 2020a). The observed resin-PFAA selectivity align with previous research results evaluating the impacts of changing the resin polymer composition and PFAAs head group using SB-AERs (Laura del Moral et al., 2020; Park et al., 2020a; Zaggia et al., 2016). The resin-PFAA selectivity has important implications on AER selection in DOC-rich waters (e.g., surface water). For example, previous literature showed lesser interference of background organic matter on PFAA removal using polystyrene than polyacrylic resin (Dixit et al., 2019; Laura del Moral et al., 2020).

### 3.4 Adsorption isotherms

#### 3.4.1 Approaches to isotherm modeling for ion-exchange

The nonlinear and linear equations and plots of each isotherm model are summarized in Table S2 in Supplementary material. To investigate the inaccuracies of the linearized curve-fitting method, the Langmuir isotherm parameters (K_L_ and q_0_) and goodness-of-fit measures for the adsorption of sulfate and nitrate were calculated using the nonlinear and four different linear forms of the Langmuir model (Table S3). Excluding A520E data, the Langmuir Type I form (linear R^2^ > 0.991) was a better fit than the Langmuir Type II form (0.600 < linear R^2^ < 0.889) for sulfate data, whereas Type II linearization provided the best fit to nitrate data (linear R^2^ > 0.995). In contrast, the A520E data were well fit by both linearization (linear R^2^ > 0.98) with a slightly better fit of Type I than Type II for nitrate and Type II than Type I for sulfate. The different outcomes are consistent with the limitations of the linearized Langmuir forms discussed in Supplementary material, all of which agreeing with AER affinity. After using the nonlinear least-squares regression method, parameters of the four linearized Langmuir forms converged to unique values varying from −169% to 62%. For instance, IRA67 (WB/PA/G/dimethyl) had ∼1.33 times the nitrate capacity of IRA458 (SB/PA/G/trimethyl) using nonlinear least-squares method but ∼3 times more with Type II linearization.

The linear models that showed decreasing average linear R^2^ values for sulfate data were Langmuir Type I (0.9940) ≈ Redlich-Peterson (RP) (0.9940) > Dubinin-Astakhov (DA) (0.9475) > Dubinin-Radushkevich (DR) (0.8571) > Langmuir Type II (0.8370) > Freundlich (0.819) > Langmuir Type III = Langmuir Type IV (0.698) (data not shown). A study on the adsorption of safranin showed a similar order of decreasing R^2^ values as RP > Langmuir Type I > Freundlich > Langmuir Type II > Langmuir Type III = Langmuir Type IV (Kumar and Sivanesan, 2005). These trends create possible misunderstanding, where adsorbates occupy a single layer within resin walls using the Langmuir Type I or the RP linearization but follow a pore filling mechanism otherwise (i.e., Langmuir Type II, III, or IV).

In summary, the nonlinear least-squares method provided a unified error distribution structure eliminating biases in the estimation of model fitting parameters and corrected abnormal values (e.g., Langmuir Type II), whereas the linearly transformed plots were conditioned by the distortion of their error structures instead of their ability to describe the theory behind each model.

#### 3.4.2 Ion-exchange behavior in single-solute systems

**Fig. 5:**
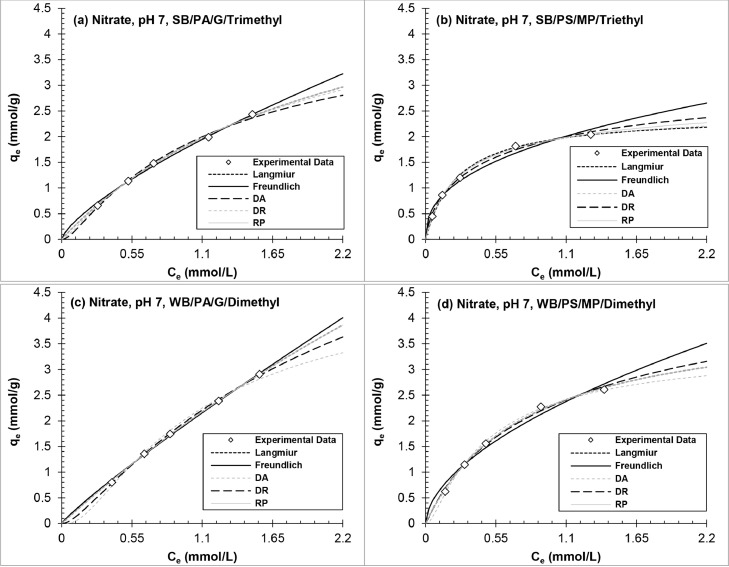
Equilibrium adsorption isotherms of nitrate onto (a, c) polyacrylic and (b, d) polystyrene AER at pH 7.

**Fig. 6:**
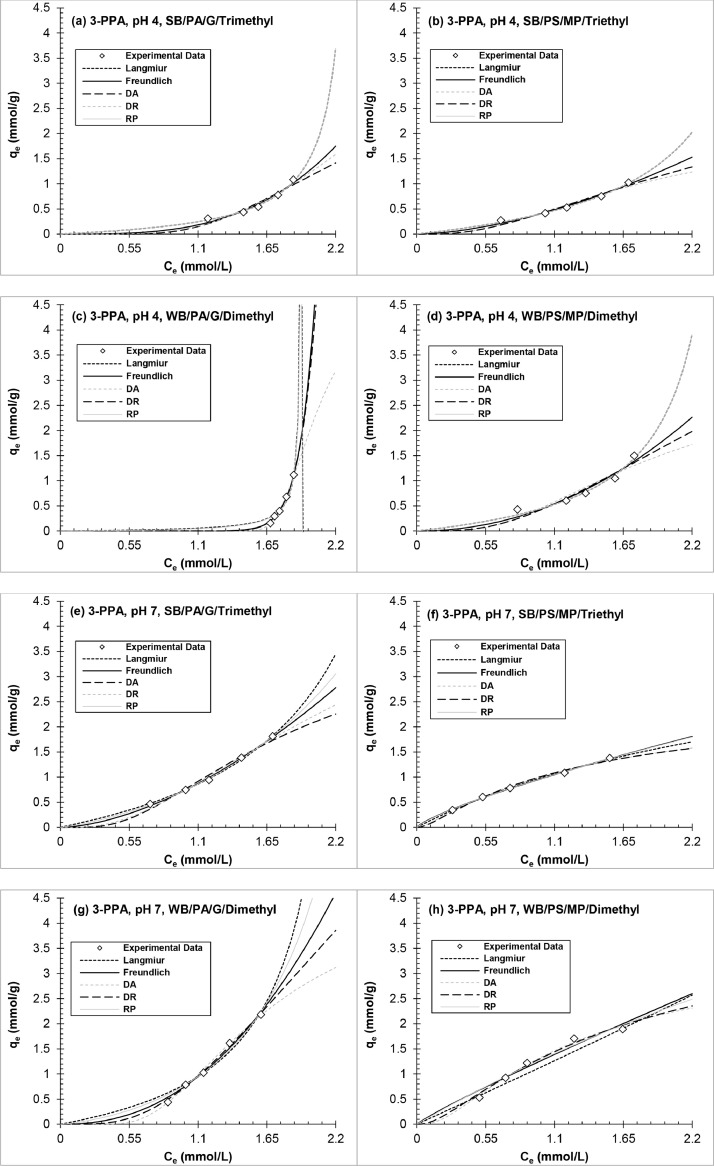
Equilibrium adsorption isotherms of 3-phenylpropionic acid (3-PPA) onto polyacrylic (left panels) and polystyrene (right panels) anion exchange resins at (a–d) pH 4 and (e–h) pH 7.

**Fig. 7:**
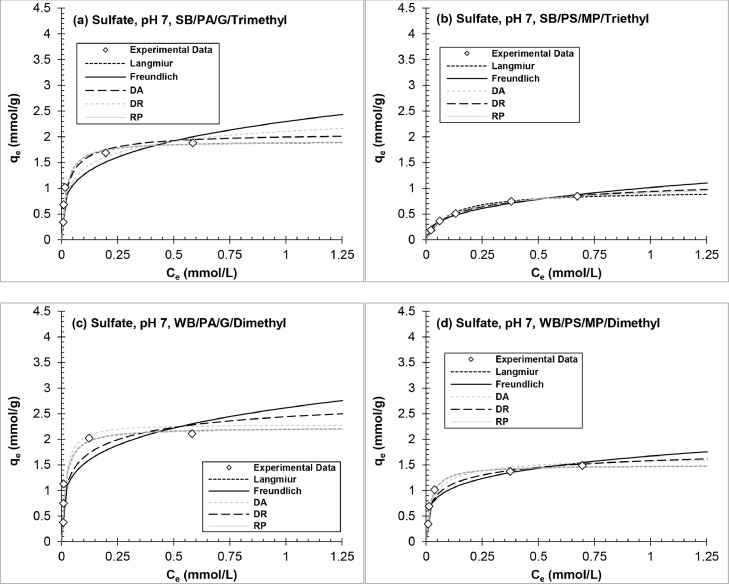
Equilibrium adsorption isotherms of sulfate onto (a, c) polyacrylic and (b, d) polystyrene AER at pH 7.

Fig. 5, Fig. 6, Fig. 7 and Figs. S2–S4 show experimental adsorption data fit to the Langmuir, Freundlich, DR, DA, and RP isotherm models using the nonlinear least-squares regression method to evaluate adsorption behavior and AER properties. The isotherm parameters are tabulated in Tables S3–S8 in Supplementary material. The following analysis excludes WB-AER at pH 10 since the resin behaves different than SB-AER under basic conditions. The order of decreasing maximum capacity for all the contaminants (*q_0_*, mmol/g) determined from the Langmuir and DR models was IRA67 (WB/PA/G/dimethyl) > IRA458 (SB/PA/G/trimethyl) > IRA67 (WB/PS/MP/dimethyl) > A520E (SB/PS/MP/triethyl). The trend shows higher capacity for WB-AER than SB-AER analogs and the lowest capacity for the triethyl alkylamine functional group (A520E), consistent with smaller tertiary amine than quaternary ammonium functional groups (Boyer et al., 2021b) and increasing distance between alkylamines with increasing chain length (Subramonian and Clifford, 1988). The estimation of the maximum capacity parameter (*q_0_*) is dependent on the solution condition (e.g., initial contaminant concentration, resin dose, water matrix), competing anions (Hekmatzadeh et al., 2012), and batch adsorption test procedure (Millar et al., 2015), all of which make comparison between studies difficult but provide useful insights on resin properties under same experimental conditions.

Regardless of the goodness of model fit to sulfate equilibrium data, the selectivity (1/n, R_L_) and thermodynamic (ΔG^0^, E) model parameters all agreed on the selectivity sequence for each resin, where selectivity increased with decreasing R_L_, 1/n, ΔG^0^ and increasing E_DR_ and E_DA_ values. A520E (SB/PS/MP/triethyl) had significantly lower selectivity for sulfate compared to IRA96 (WB/PS/MP/dimethyl), which showed equally high selectivity than remaining AERs. A520E and IRA96 have same polymer composition but differ in the identity of functional group (i.e., triethylamine vs. dimethylamine). Although the removal results of previous research and in Fig. 1c had demonstrated increasing sulfate adsorption with increasing resin polarity and decreasing spacing of functional groups (Hu et al., 2016), the model data in this work are complementary suggesting much greater impact of AER site spacing than polymer composition.

Figs. S5–S7 show binary exchange plots for chloride-form AER in single-solute systems. The contaminant is more preferred by AER over the mobile counterion (i.e., Cl^−^) as the distance of data points above the 1:1 line increases. The general order of decreasing selectivity for each AER at pH 4 and 7 were as follows:

• IRA458 (SB/PA/G/trimethyl) SO_4_^2−^ > NO_3_^−^ ≈ Cl^−^ > 3-PPA (pH 7) > 3-PPA (pH 4)
• A520E (SB/PS/MP/triethyl) NO_3_^−^ > SO_4_^2−^ > 3-PPA (pH 7) ≈ Cl^−^ > 3-PPA (pH 4)
• IRA67 (WB/PA/G/dimethyl) SO_4_^2−^ > NO_3_^−^ ≈ Cl^−^ > 3-PPA (pH 7) > 3-PPA (pH 4)
• IRA96 (WB/PS/MP/dimethyl) NO_3_^−^ ≈ SO_4_^2-^ > 3-PPA (pH 7) > Cl^−^ > 3-PPA (pH 4)

The results agree with isotherm models (Fig. 5–7 and S2–S4) and normalized removal data (Fig. 1). Looking collectively at adsorption isotherms, model parameters, and binary exchange plots for the nitrate and sulfate equilibrium experiments, all plots were the same between pH 4 and pH 7 with relative difference <10% for model parameters. Sulfate showed the highest affinity among the contaminants for all AERs except A520E resin. Finally, the trends for IRA458 (SB/PA/G/trimethyl) and IRA67 (WB/PA/G/dimethyl) support the discussion in Section 3.2 on the negligible impact of solution pH and AER basicity on resin selectivity at pH ≤7.

To further investigate the ion-exchange process, the equivalent concentration of contaminant removed from solution (meq/L) was plotted as a function of chloride ions released from the resin (meq/L) (Figs. S8–10). Most data points were on the 1:1 line (i.e., stoichiometric exchange) indicating that the removal of nitrate, sulfate, and 3-PPA is predominantly through electrostatic interactions. The reason for the higher release of chloride than the amount of contaminant removed by WB-AER was due to the deprotonation of amine groups in unbuffered test water accompanied by a release of the mobile counterion (i.e., chloride) (see Section 3.1). The results of nitrate and sulfate further justify the observed difference between SB- and WB-AER experiments since inorganic contaminants could only bind electrostatically to the resin. Most importantly, the uptake of 3-PPA by A520E (SB/PS/MP/triethyl) resulting in stoichiometric release of chloride at pH 7 (Fig. S9c), pH 10 (Fig. S10b), and to a lesser extent at pH 4 (Fig. S8c) implies that electrostatic interactions are necessary for removal regardless of the hydrophobic character of the system, which rather contributes to the selectivity between the solute and the resin (Landry et al., 2015). The results are consistent with previous studies evaluating the ion-exchange stoichiometry using polystyrene resin for the removal of large DOC compounds (Boyer et al., 2008; Walker and Boyer, 2011) and HIOCs (Landry and Boyer, 2013; Li and SenGupta, 1998; 2004).

In terms of nonlinear R^2^ and ARE values, the order of decreasing two-parameter model fit to sulfate equilibrium data was DA ≈ DR >> Langmuir > Freundlich, while nitrate equilibrium data was equally well-fit by all isotherm models (R^2^ >0.96; ARE <10%), suggesting that ion-exchange of sulfate and nitrate were best represented by intraparticle diffusion rather than the layer-by-layer theory. This approach was supported in a previous study for various inorganic counterion/solute ion-exchange systems including Cl^−^, NO_3_^−^, HCO_3_^−^, SO_4_^2−^, and OH^−^ (Dron and Dodi, 2011a). The DA and DR models suggest adsorption by AER to be based on the average free energy of the resin-solute-solution system with electrostatic interactions as the primary mechanism of removal. For example, Li and SenGupta (1998) showed favorable adsorption when the hydrophobic character of the solute is closest to that of adsorbent (polyacrylic vs. polystyrene) than of solution (water vs. cosolvent).

### 3.5 Implications of adsorption mechanisms for WB-AER treatment

**Fig. 8:**
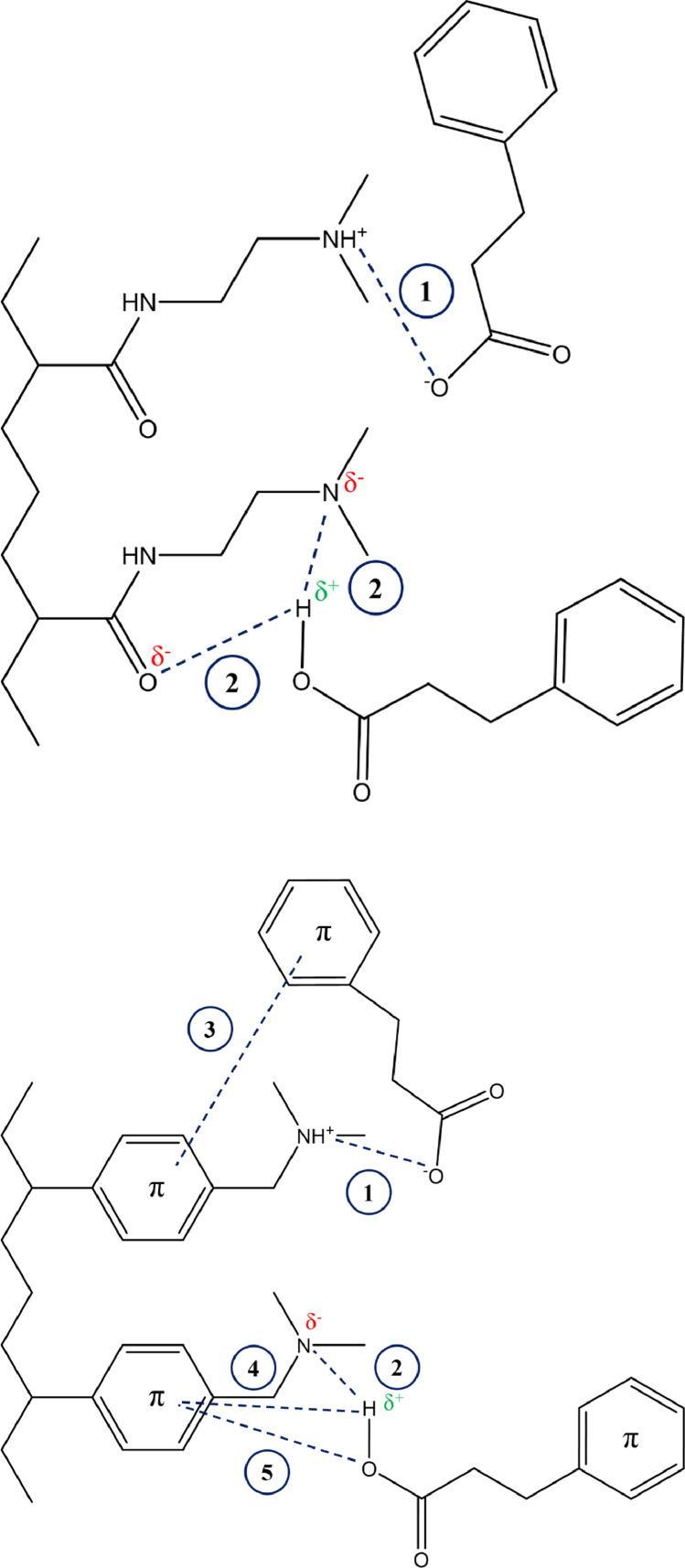
Schematic of different interactions between unprotonated (top) or protonated (bottom) 3-phenylpropionic (3-PPA) acid molecules and dimethylamine groups of (a) IRA67 and (b) IRA96 resins. Dashed lines indicate (1) electrostatic interactions, (2) dipole-dipole bonds (3) π-π lateral bonds, (4) Yoshida hydrogen bonds, and (5) *n-*π bonds (Tran et al., 2017a, 2017b).

Fig. 8 shows possible interactions contributing to hydrophobic contaminant (e.g., 3-PPA, PFAA) removal by WB-AER. These include (1) electrostatic interactions and non-electrostatic interactions, namely hydrophobic attractions, and van der Waals forces such as (2) dipole-dipole interactions, (3) π-π bonding, (4) Yoshida hydrogen bonding, and (5) *n-*π bonding (Tran et al., 2017b).

#### 3.5.1 WB/PA/G/dimethyl resin (IRA67)

The close-packed methyl amine functional group and polyacrylic composition of IRA67 (WB/PA/G/dimethyl) impart greater sulfate selectivity and higher ion-exchange capacity among all the resins. In addition, the IRA67 resin showed the greatest reduction in removal efficiency under highly basic conditions, which implied effective regeneration with dilute solution of NaOH. At pH > pK_a_, electrostatic interactions are substituted by 15–20 times weaker dipole-dipole hydrogen bonds (Fig. 8a) occurring between the deprotonated nitrogen atom of the resin functional group and polarized hydrogen atoms of the solute head group (e.g., hydroxyl, carbonyl) (Bolto et al., 2002; Greluk and Hubicki, 2011). Sulfates are known competitors for ion-exchange sites (Song et al., 2012) and have shown to reduce the resin capacity for nitrates (Hekmatzadeh et al., 2012) and PFAS, such as short-chain PFAAs (Zeng et al., 2020) and the more selective PFOA (Yang et al., 2018), PFHxS (Maimaiti et al., 2018), PFOS (Deng et al., 2010), and F-53B (Gao et al., 2017). The semiconductor industry requires high amounts of soluble sulfate-based organic chemicals for chip manufacturing while also discharging high levels of PFOS (i.e., hundreds μg/L) (Lin et al., 2009). Both presume using reverse osmosis and/or nanofiltration for PFAS treatment in sulfate-rich waters, which is problematic due to scaling by calcium sulfate and barium sulfate (MacAdam and Jarvis, 2015). Therefore, a pretreatment step using WB/PA/G/dimethyl resin (e.g., IRA67) could be a cost-effective strategy to increase membrane longevity.

#### 3.5.2 WB/PS/MP/dimethyl resin (IRA96)

The WB/PS/MP/dimethyl resin (IRA96) demonstrated high affinity for nitrate, 3-PPA, and PFAAs due to the nonpolar polystyrene composition of the resin, which has the potential to form π-bonds (see Fig. 8b) between aromatic carbons of the resin matrix and the solute (Li and SenGupta, 2004; Tran et al., 2017a). Transferring the solute from water (polar solvent) is more favorable with increasing hydrophobicity of solute and/or resin but would require a regeneration solution with nonpolar character (e.g., methanol) for effective desorption (Du et al., 2014; Landry and Boyer, 2013). The next step in this work is to evaluate the reuse of PFAAs-laden IRA96 resin considering salt/solvent composition of the regeneration solution. In addition, the high buffering capability of IRA96 observed in this study and in a previous study (Zhang et al., 2014) would provide a more selective removal of PFAAs. For example, PFAAs removal from typically acidic (pH ∼3) chromium plating wastewater was shown to be less effective in the presence of competing chromate anions (Deng et al., 2010; Gao et al., 2017; Hu et al., 2016) with a decrease in chromium removal as pH increased over the range 2–6 (Bajpai et al., 2012; Edebali and Pehlivan, 2010; Wang et al., 2015). The use of IRA96 would thus play important role in PFAA uptake.

## 5 Conclusions

• At neutral pH, contaminant removal was not influenced by the basicity of the resin functional groups. This indicated the potential for WB-AER to be used in place of SB-AER in water and wastewater treatment applications.
• WB- and SB-AER of polystyrene composition were proven most effective for PFAA removal with a greater selectivity for PFSAs than PFCAs and for PFOS than remaining PFAAs. To minimize the impact of competing sulfate and carbonate anions on PFAA adsorption, AERs with large alkylamine functional groups and polystyrene composition are suggested. These conclusions are limited to synthetic solutions containing the six PFAAs tested in this work. Future research should evaluate real test waters rich in competing organic matter and explore WB-AER with functional groups that are more selective for monovalent anions to extend its use in PFAA-impacted water.
• The results at pH >10 demonstrated the protonation of tertiary amine resin functional groups to be reversible and pH-dependent. This implies the amenable regeneration of WB-AER using alkaline solutions with lower impacts than organic cosolvent regeneration.
• WB polystyrene IRA96 resin maintained complete removal of PFAAs at low concentration (μg/L), suggesting electrostatic binding between PFAA and remaining protonated amine functional groups on the resin. Further research should focus on continuous-flow column experiments to explore the trade-offs in both PFAAs removal by WB/polystyrene and regeneration by WB/polyacrylic AERs.
• The nonlinear least-squares regression is essential when applying the Langmuir model to avoid biases from the four linearized forms, whereas the remaining two-parameter isotherm models were not affected by the regression method. All isotherms agreed on the greater capacity of WB-AERs than SB-AERs with same polymer composition implying benefit for operation and treatment. Relying on goodness-of-fit measures only is not enough to determine the adsorption mechanism and could be misleading, as two isotherms with conflicting theories (e.g., Langmuir, Freundlich) might fit equilibrium data similarly. Therefore, qualitative rather than quantitative analysis should be considered to identify potential trends and three-parameter hybrid models such as Redlich-Peterson are recommended given the broader applicability.

## CRediT authorship contribution statement

**Christian Kassar:** Investigation, Formal analysis, Visualization, Writing – original draft. **Cole Graham:** Investigation. **Treavor H. Boyer:** Conceptualization, Writing – review & editing, Funding acquisition.

## Declaration of Competing Interest

The authors declare that they have no known competing financial interests or personal relationships that could have appeared to influence the work reported in this paper.

## Data Availability

Data will be made available on request.

## Appendix Supplementary materials

Image, application 1
