# Supplementary material for: Removal of perfluoroalkyl acids and common drinking water contaminants by weak-base anion exchange resins: Impacts of solution pH and resin properties
Source: Water Res X. 2022 Nov 2;17:100159. doi: 10.1016/j.wroa.2022.100159 (PMC9650071; doi:10.1016/j.wroa.2022.100159)
Supplement: Supplementary file 1 [file mmc1.docx]

**Removal of perfluoroalkyl acids and common drinking water contaminants by weak-base anion exchange resins: Impacts of solution pH and resin properties**

**Christian Kassar, Cole Graham, and Treavor H. Boyer***

**School of Sustainable Engineering and the Built Environment (SSEBE)**

**Arizona State University**

**PO Box 873005, Tempe, AZ 85287-3005, USA**

***Corresponding author.**

**Tel.: 1-480-965-7447**

**E-mail addresses: thboyer@asu.edu (T.H. Boyer)**

**Submitted to**

***Water Research***

**Adsorption isotherms**

Adsorption isotherms portray the dependence of analyte concentration in the resin phase (q_e_) on the aqueous concentration (C_e_) at equilibrium (Helfferich, 1995). Langmuir, Freundlich, Dubinin-Radushkivich (DR) and Dubinin-Astakhov (DA) models were chosen and determined using the nonlinear least-squares (NLS) regression method in Rstudio (Version 1.4.1717). Parameters were first obtained plotting linear forms of each isotherm model to initiate values for the NLS function. To assess model fits of experimental data, the correlation coefficient (R^2^), the average relative error (ARE), the sum of squared errors (SSE), the root mean squared error (RMSE), and the chi-squared (χ^2^) were calculated using the metrics RStudio package (see Eqs. S12-S16). R^2^, ARE and χ^2^ were used to compare the fitting of multiple models between all equilibrium data. SSE and RMSE are not normalized by the concentration in the resin phase, qe. (i.e., dimensionless) and were thus used to identify the best-fit model for each equilibrium experiment only. In the adsorption isotherm graphs, qe (mmol/g), the contaminant concentration in the resin phase, is plotted against Ce (mmol/L), the aqueous contaminant concentration, at equilibrium. Isotherm models are chosen to evaluate the model approach that best describes adsorption mechanism. These include thermodynamic, kinetic, and potential theory considerations with each isotherm model derived in terms of at least one approach (Foo and Hameed, 2010).

***Langmuir isotherm model***

The Langmuir isotherm (Eq. S1) implies that the adsorbent has limited number of sites with equal and constant adsorption energy and homogeneous structure (Chan et al., 2012). Aqueous contaminants have similar thermodynamic character (i.e., enthalpy, activation energy of adsorption), which suggests equal preference for accessible sites (Tran et al., 2017). Once adsorbed, only a one-molecule layer can be formed on the adsorbent, with no lateral transport on the surface and interactions between adsorbates (Foo and Hameed, 2010).

$q_{e}=\frac{q_{0}K_{L}C_{e}}{1+K_{L}C_{e}}$ (S1)

Where q_0_ (mmol/g) is the maximum adsorption capacity of the resin and K_L_ (L/mmol) is the Langmuir coefficient related to the affinity between the adsorbent and adsorbed species. Further useful parameters such as the dimensionless separation factor (R_L_) (Eq. S2) and the change in Gibbs free energy (ΔG^0^) (Eq. S3) are calculated as follows:

$R_{L}=\frac{1}{1+K_{L}C_{0}}$ (S2)

${\Delta G}^{0}=-RT\ln K_{L}$ (S3)

Where T (K) is the temperature (average of 275.41 K in this study), R is the ideal gas constant (8.314 J/K mol). The extent of isotherm favorability is portrayed by R_L_, whereby values between 0 and 1 represents suitable fit while being unfavorable above 1 (Namasivayam and Ranganathan, 1995). Negative values of the thermodynamic parameter ΔG^0^ (kJ/mol) suggest favorable and spontaneous adsorption of aqueous contaminants, otherwise unfavorable and nonspontaneous (Dron and Dodi, 2011). More than four expressions for the linearized Langmuir equation are available, each yielding different R^2^ and fitted parameter values (Bolster and Hornberger, 2007). The form with the highest R^2^ was used to determine starting parameters for the NLS function.

*Linearized forms of the Langmuir isotherm model*

The limitations of using the linearized forms of the Langmuir equation (Table S1) include misestimation of the Langmuir selectivity and capacity parameters and providing a deceiving fit to experimental data with Type I (Hanes-Woolf) and Type II (Lineweaver-Burk) forms most commonly employed (Kumar and Sivanesan, 2005).

**Langmuir Type I (Hanes Woolf):** C_e_/q_e_ (y-axis) and C_e_ (x-axis) are strongly interdependent and could provide misleading deduction regarding the adsorption mechanism falsely representing the Langmuir model (Bolster and Hornberger, 2007). The axial setting of this form provides the lowest error distribution and gives the impression of a favorable fit to experimental data due to high R^2^ values. The strong correlation between the dependent variables was designated as “mathematical artifact” (El-Khaiary, 2008).

**Langmuir Type II (Lineweaver-Burke):** Disadvantageous when q_e_ values approach zero since 1/q_e_ values become extremely sensitive to small variations of 1/C_e_ (x-axis). This form fails to portray very high removal at low concentrations which could lead to highest deviation in model parameter estimation.

**Langmuir Type III (Eadie-Hoffsiee) and Type IV (Scatchard):** Both forms are associated to the same error distribution structure due to their identical x and y correlation (i.e., q_e_/C_e_ and q_e_) and thus provide the same linear R^2^ values. Lower K_L_ values are compensated by higher values of q_0_ despite the same fit to all equilibrium data (see Table S2).

***Freundlich isotherm model***

As opposed to the semi-empirical Langmuir model (Langmuir, 1916), the Freundlich isotherm is an empirical model (Eq. S4) (Freundlich, 1906), which assumes bilayer formation of solutes on the surface of the adsorbent, heterogeneous sites, and non-ideal systems. The isotherm suggests potential for unlimited adsorbent capacity, translated by its shape not exhibiting a plateau, with the thermodynamic approach not accounted for (Ahmed and Dhedan, 2012). Hence, the Freundlich model lacks the ability to describe saturation effects at high analyte concentration while not converging to the Henry’s law at trace levels (Chan et al., 2012; Dron and Dodi, 2011; Foo and Hameed, 2010).

$q_{e}=K_{F}C_{e}^{1/n}$ (S4)

Where K_F_ (mmol/g)/(mmol/L)^1/n^ and 1/n (dimensionless) are the Freundlich constants representative of adsorption intensity and the extent of surface heterogeneity or selectivity respectively. Complementary to the R_L_ parameter of the Langmuir model, 1/n was used to evaluate the adsorption nature, where adsorption is favorable, linear, and unfavorable for 1/n values below, equal, and above unity respectively (Tran et al., 2017).

***Dubinin-Radushkevich (DR) and Dubinin-Astakhov (DA) isotherm models***

The DR and DA empirical models assume pore filling adsorption onto microporous solids (Dubinin, 1947) rather than the layer-by-layer mechanism of previous models (i.e., Langmuir and Freundlich) (Inglezakis, 2007). The Dubinin isotherms are based on the Polanyi potential theory of adsorption expressed in Eq. 6 by the temperature dependent Polanyi potential (*ε*) parameter.

$\varepsilon=RT\ln(1+\frac{1}{C_{e}})$ (S5)

Where C_e_ (g/g) is the concentration of analyte at equilibrium. Here, the non-solubility-normalized *ε* highly relates to the negative value of the Langmuir thermodynamic change in free energy parameter (Eq. S3) (Inglezakis, 2007). The nonlinear DR and DA parameters are calculated by fitting equilibrium data to Eq. S6 and Eq. S7 respectively. of contaminants from the aqueous to the surface of the adsorbent

$q_{e}=q_{0} exp\left[ -\left( \frac{\varepsilon}{E\sqrt{2}} \right)^{2} \right]$ (S6)

$q_{e}=q_{0} exp\left[ -\left( \frac{\varepsilon}{E\sqrt{2}} \right)^{n_{D}} \right]$ (S7)

Where *q_e_* and *q_0_* are the equilibrium contaminant concentration in the solid phase and the maximum adsorbent capacity (g/g) respectively. *E* is the adsorption energy (J/mol), defined as energy needed for the contaminant to partition between the aqueous and solid phases from infinity (Onyango et al., 2004; Özcan et al., 2005), and n_D_ is the heterogeneity parameter (dimensionless). The magnitude of E indicates the type of adsorption (i.e., chemical or physical) and is typically useful for the removal of organic ionizable compounds (OICs) by AER (Fu et al., 2008; Milmile et al., 2011; Özcan et al., 2005; Tran et al., 2017). For the DR isotherm n_D_ = 2 (i.e., non-ideal) while higher values (n_D_ > 3) impart homogeneous pore structure (Dron and Dodi, 2011; Inglezakis, 2007). Both isotherms were treated as two-parameter models, whereby *E* and *q_0_*, and *E* and *n_D_* were obtained through nonlinear regression for DR and DA isotherms respectively while *q_0_* was determined experimentally for the DA isotherm as recommended elsewhere (Dron and Dodi, 2011; Landry et al., 2015).

***Redlich-Peterson (RP) isotherm model***

The RP is a three-parameter empirical model able to predict ideal and non-ideal adsorption at a wide range of concentrations (Chen et al., 2014; Foo and Hameed, 2010). As shown in Eq. S8, based on the dimensionless parameter, α (0-1), and C_e_ values, the hybrid RP model converts to the Langmuir (Eq. S9), the Freundlich (Eq. S10), and the Henry’s law (Eq. S11) equations.

$q_{e}=\frac{K_{RP}C_{e}}{1+b_{RP}C_{e}^{\alpha}}$ (S8)

$q_{e}=\frac{K_{RP}C_{e}}{1+b_{RP}C_{e}}=\frac{q_{0}K_{L}C_{e}}{1+K_{L}C_{e}}$ (S9)

$q_{e}=\frac{K_{RP}}{b_{RP}}{C_{e}}^{1-\alpha}=K_{F}C_{e}^{1/n}$ (S10)

$q_{e}=\frac{K_{RP}C_{e}}{1+b_{RP}}=H_{YC}C_{e}$ (S11)

Where $K_{RP}$ (L/mmol) and $b_{RP}$ (L/mmol)^α^ are the RP constants, α (dimensionless) is the RP exponent, $K_{L}$ and $q_{0}$ are the Langmuir constants, $K_{F}$ and $1/n$ are the Freundlich constants, and $H_{YC}$ is the dimensionless Henry’s law constant. Briefly, the RP reduces to the Langmuir equation (Eq. S9) when α approaches 1, where $K_{RP}$ and $b_{RP}$ equal $q_{0}K_{L}$ and $K_{L}$ respectively (Chen et al., 2014; Foo and Hameed, 2010); it reduces to the Freundlich equation (Eq. S10) at large contaminant concentration (Jossens et al., 1978; Radke and Prausnitz, 1972) where ${K_{RP}}/{b_{RP}}$ equals $K_{F}$ and $1-\alpha$ equals $1/n$; and is in accordance with the Henry’s law (Eq. S11) whenever α is close to 0 (Howe et al., 2012). Considering the mathematical complexities of a three-parameter model, the linearized RP form (see Table S1) is to be solved by either minimizing the SSE or maximizing the R^2^ error functions to initiate values for the nonlinear model parameters (Allen et al., 2003).

**Goodness-of-fit measures**

Mathematical error functions from current literature were explored to determine the best-fit model for ion-exchange systems. All the following equations (Eqs. S1-5) could both be applied to linear and nonlinear data except for chi-squared (χ^2^) being specific to the nonlinear regression method (Tran et al., 2017).

$R^{2}=1-\frac{\sum_{i=1}^{n} {(Y-\hat{Y})}^{2}}{\sum_{i=1}^{n} {(Y-\overline{Y})}^{2}}$ (S12)

$ARE =\frac{100}{n}\sum_{i=1}^{n} \frac{\left| Y-\hat{Y} \right|}{Y}$ (S13)

$SSE=\sum_{i=1}^{n} {(Y-\hat{Y})}^{2}$ (S14)

$RMSE=\sqrt{\frac{1}{n}\sum_{i=1}^{n} {(Y-\hat{Y})}^{2}}$ (S15)

$\chi^{2}=\sum_{i=1}^{n} \frac{{(q_{e,meas}-q_{e,calc})}^{2}}{q_{e,calc}}$ (S16)

Where $Y$, $\overline{Y}$, and $\hat{Y}$ are the ordinate data, mean of ordinate data, and the ordinate data achieved from the linear or the nonlinear of the model, respectively. Accordingly, for nonlinear equilibrium adsorption models, $Y$, $\overline{Y}$ and $\hat{Y}$ are the measured (q_e,meas_; meq/g), the mean of measured ($\overline{q_{e,meas}}$; meq/g), and the calculated (q_e,calc_; meq/g) equivalent concentrations of contaminants in the resin phase, respectively.

The coefficient of determination R^2^ (Eq. S12) is a widely used statistical measure that compares the variance of independent variables (i.e., q_e,meas_ and q_e,calc_ ) to the variance about the mean of dependent variables (i.e., q_e,meas_ and $\overline{q_{e,meas}}$) (Foo and Hameed, 2010). Values closer to 1 indicate a better fit of a model to experimental data. The average relative error (ARE) (Eq. S13) reflects on the degree of bias in the predicted model results. Both these error functions are used to evaluate model fitting for all solute-resin pairs (Dron and Dodi, 2011; Hu et al., 2016; Landry et al., 2015). The SSE and RMSE (Eqs. S14 and S15) reflect on the variance of independent variables and are good indicators of model fitting to experimental data where isotherms provide a better fit when SSE and RMSE values are closer to 0 (Kinniburgh, 1986). However, both these functions could only be used to compare the fit of different models to one equilibrium experiment as the values are not normalized (Dron and Dodi, 2011) and become more sensitive to variation at the highest end of the curve (i.e., high C_e_ values) (Allen et al., 2003). χ^2^ (Eq. 16) is specific to nonlinear regression only, where high χ^2^ values indicate an underestimation or an overestimation of the calculated concentration in the resin phase, while conforming to the experimental data when approaching 0 (Tran et al., 2017).

**Table S1.** Physicochemical properties and initial concentrations of PFAA tested in this study.

| Acronym, Number of Carbon | Structure^a^ | Molecular Weight (g/mol) | Log D_ow_^b^ (pH=7) | Equivalent Concentration (μeq/L)^c^ |
| --- | --- | --- | --- | --- |
| PFCA | | | | |
| PFBA, C4 |  | 214 | -1.22 | 0.374 |
| PFHxA, C6 |  | 314.1 | 0.18 | 0.255 |
| PFOA, C8 |  | 414.1 | 1.58 | 0.193 |
| PFSA | | | | |
| PFBS, C4 |  | 300.1 | 0.25 | 0.267 |
| PFHxS, C6 |  | 400.1 | 1.65 | 0.200 |
| PFOS, C8 |  | 500.1 | 3.05 | 0.160 |

^a^ ChemDraw Professional (20.1.1). PFAA shown in the dissociated form.
^b^ (Park et al., 2020; Zeng et al., 2020).
^c^ Initial concentration of each PFAAs was C_0_ = 80 μg/L. Total concentrations were $\sum\mathrm{PFAAs}$ = 480 μg/L (1.45 μeq/L).

**Table S2.** Adsorption isotherm models and corresponding linear forms and plots used to obtain starting values for the nonlinear-optimization method.

| Adsorption Isotherm | Nonlinear Form | Linear Form | Plot |
| --- | --- | --- | --- |
| Langmuir Type I (Hanes-Woolf) | $q_{e}=\frac{q_{0}K_{L}C_{e}}{1+bC_{e}}$ | $\frac{C_{e}}{q_{e}}=\frac{1}{K_{L}q_{0}}+\frac{1}{q_{0}}C_{e}$ | $\frac{C_{e}}{q_{e}} vs C_{e}$ |
| Langmuir Type II (Lineweaver-Burke) |  | $\frac{1}{q_{e}}=\frac{1}{q_{0}}+\frac{1}{K_{L}q_{0}}\frac{1}{C_{e}}$ | $\frac{1}{q_{e}} vs \frac{1}{C_{e}}$ |
| Langmuir Type III (Eadie-Hoffsiee) |  | $q_{e}=q_{0}-\frac{1}{K_{L}}\frac{q_{e}}{C_{e}}$ | $q_{e} vs \frac{q_{e}}{C_{e}}$ |
| Langmuir Type IV (Scatchard) |  | $\frac{q_{e}}{C_{e}}=q_{0}-K_{L}q_{e}$ | $\frac{q_{e}}{C_{e}} vs q_{e}$ |
| Freundlich | $q_{e}=K_{F}C_{e}^{1/n}$ | $\log{(q}_{e})=\log{(K}_{F})+\frac{1}{n}\log C_{e}$ | $\log{(q}_{e}) vs \log{(C}_{e})$ |
| Dubinin-Astakhov | $q_{e}=q_{0}exp(-\left( \frac{\varepsilon}{E\sqrt{2}} \right)^{n_{D}})$ | $\ln(ln \left( q_{0} \right)-\ln\left( q_{e} \right))=-n_{D}\ln\left( E\sqrt{2} \right)+n_{D}\ln(\varepsilon)$ | $ln( \ln{(q}_{e})) vs \ln(\varepsilon)$ |
| Dubinin- Radushkevich | $q_{e}=q_{0}exp(-\left( \frac{\varepsilon}{E\sqrt{2}} \right)^{2})$ | $\ln(q_{e})=\ln(q_{0})-\frac{1}{2E^{2}}\varepsilon^{2}$ | $\ln{(q}_{e}) vs \varepsilon^{2}$ |
| Redlich-Peterson^a^ | $q_{e}=\frac{K_{RP}C_{e}}{1+b_{RP}C_{e}^{\alpha}}$ | $\ln(K_{RP}\frac{C_{e}}{q_{e}}-1)=\ln(b_{RP})+\alpha\ln(C_{e})$ | $\ln(K_{RP}\frac{C_{e}}{q_{e}}-1) vs \ln(C_{e})$ |

**Table S3.** Nonlinear and linear Langmuir isotherm and goodness-of-fit model parameters for the single-analyte adsorption of (a) nitrate and (b) sulfate on AER at pH 4, 7 and 10. Nonlinear parameter values were determined using the least-squares regression method in RStudio. Linear parameter values were determined plotting corresponding linear forms of the Langmuir equation.

**(a) Nitrate**

| Resin/pH | Langmuir Equation | K_L_ (L/mmol) | q_0_ (mmol/g) | Linear R^2^ | Nonlinear R^2^ | ARE (%) | SSE (mmol^2^/g^2^) | RMSE (mmol/g) |
| --- | --- | --- | --- | --- | --- | --- | --- | --- |
| IRA 458/4 | Linear Type I | 0.29 | 9.29 | 0.8762 | 0.988 | 2.291 | 0.0938 | 0.0791 |
|  | Linear Type II | 0.27 | 9.93 | 0.9985 | 0.9877 | 2.412 | 0.0963 | 0.0801 |
|  | Linear Type III | 0.33 | 8.49 | 0.8309 | 0.9876 | 2.436 | 0.0973 | 0.0805 |
|  | Linear Type IV | 0.27 | 9.89 | 0.8309 | 0.9877 | 2.399 | 0.0960 | 0.0800 |
|  | Nonlinear | 0.30 | 9.08 | - | 0.9881 | 2.378 | 0.0934 | 0.0789 |
| A520E/4 | Linear Type I | 4.28 | 2.48 | 0.9978 | 0.9964 | 3.390 | 0.0200 | 0.0366 |
|  | Linear Type II | 5.41 | 2.23 | 0.9971 | 0.9801 | 4.082 | 0.1106 | 0.0859 |
|  | Linear Type III | 4.83 | 2.37 | 0.9788 | 0.9933 | 3.296 | 0.0372 | 0.0498 |
|  | Linear Type IV | 4.73 | 2.39 | 0.9788 | 0.9944 | 3.305 | 0.0313 | 0.0457 |
|  | Nonlinear | 4.17 | 2.49 | - | 0.9968 | 3.469 | 0.018 | 0.0346 |
| IRA 67/4 | Linear Type I | 0.15 | 16.05 | 0.7654 | 0.9941 | 2.394 | 0.0455 | 0.0551 |
|  | Linear Type II | 0.09 | 25.65 | 0.9967 | 0.9869 | 2.602 | 0.1011 | 0.0821 |
|  | Linear Type III | 0.20 | 12.42 | 0.6797 | 0.9946 | 2.533 | 0.0416 | 0.0527 |
|  | Linear Type IV | 0.13 | 17.43 | 0.6797 | 0.9933 | 2.401 | 0.0518 | 0.0588 |
|  | Nonlinear | 0.19 | 12.58 | - | 0.9952 | 2.525 | 0.037 | 0.0497 |
| IRA 96/4 | Linear Type I | 1.07 | 4.65 | 0.9962 | 0.9984 | 1.267 | 0.0135 | 0.0300 |
|  | Linear Type II | 1.03 | 4.78 | 0.9992 | 0.998 | 1.247 | 0.0169 | 0.0336 |
|  | Linear Type III | 1.06 | 4.68 | 0.9917 | 0.9984 | 1.252 | 0.0138 | 0.0303 |
|  | Linear Type IV | 1.05 | 4.71 | 0.9917 | 0.9983 | 1.251 | 0.0142 | 0.0308 |
|  | Nonlinear | 1.09 | 4.62 | - | 0.9984 | 1.291 | 0.0133 | 0.0298 |
| IRA 458/7 | Linear Type I | 0.45 | 5.96 | 0.8417 | 0.9715 | 3.746 | 0.1689 | 0.1061 |
|  | Linear Type II | 0.41 | 6.41 | 0.9948 | 0.971 | 3.570 | 0.1719 | 0.1071 |
|  | Linear Type III | 0.51 | 5.45 | 0.7792 | 0.9702 | 4.129 | 0.1764 | 0.1084 |
|  | Linear Type IV | 0.40 | 6.56 | 0.7792 | 0.9704 | 3.576 | 0.1752 | 0.1081 |
|  | Nonlinear | 0.46 | 5.93 | - | 0.9716 | 3.706 | 0.168 | 0.1058 |
| A520E/7 | Linear Type I | 4.09 | 2.42 | 0.9976 | 0.994 | 3.868 | 0.0313 | 0.0457 |
|  | Linear Type II | 4.99 | 2.20 | 0.9816 | 0.9798 | 5.332 | 0.1052 | 0.0837 |
|  | Linear Type III | 4.59 | 2.31 | 0.9560 | 0.9906 | 4.423 | 0.0491 | 0.0572 |
|  | Linear Type IV | 4.39 | 2.36 | 0.9560 | 0.9927 | 4.112 | 0.0381 | 0.0504 |
|  | Nonlinear | 3.97 | 2.44 | - | 0.9942 | 4.051 | 0.0301 | 0.0448 |
| IRA 67/7 | Linear Type I | 0.10 | 21.97 | 0.6817 | 0.9954 | 2.325 | 0.038 | 0.0504 |
|  | Linear Type II | 0.05 | 38.6 | 0.9971 | 0.9908 | 2.547 | 0.0763 | 0.0713 |
|  | Linear Type III | 0.15 | 15.01 | 0.6055 | 0.9947 | 2.414 | 0.0436 | 0.0539 |
|  | Linear Type IV | 0.09 | 23.59 | 0.6055 | 0.9951 | 2.336 | 0.0408 | 0.0522 |
|  | Nonlinear | 0.13 | 17.58 | - | 0.9959 | 2.250 | 0.0343 | 0.0478 |
| IRA 96/7 | Linear Type I | 1.23 | 4.18 | 0.9691 | 0.985 | 3.579 | 0.1186 | 0.0889 |
|  | Linear Type II | 0.99 | 4.81 | 0.9969 | 0.9744 | 2.934 | 0.2026 | 0.1162 |
|  | Linear Type III | 1.16 | 4.33 | 0.9417 | 0.9845 | 3.259 | 0.1232 | 0.0906 |
|  | Linear Type IV | 1.09 | 4.49 | 0.9417 | 0.9824 | 3.111 | 0.1394 | 0.0964 |
|  | Nonlinear | 1.28 | 4.12 | - | 0.9856 | 3.668 | 0.1144 | 0.0873 |
| IRA 458/10 | Linear Type I | 0.3 | 7.23 | 0.9667 | 0.9969 | 1.693 | 0.0169 | 0.0336 |
|  | Linear Type II | 0.28 | 7.81 | 0.9987 | 0.9959 | 1.497 | 0.0225 | 0.0387 |
|  | Linear Type III | 0.31 | 7.11 | 0.9458 | 0.9970 | 1.741 | 0.0165 | 0.0332 |
|  | Linear Type IV | 0.29 | 7.43 | 0.9458 | 0.9967 | 1.613 | 0.0181 | 0.0348 |
|  | Nonlinear | 0.32 | 6.89 | - | 0.9971 | 1.831 | 0.016 | 0.0327 |
| A520E/10 | Linear Type I | 3.84 | 2.76 | 0.9983 | 0.9971 | 3.291 | 0.02 | 0.0365 |
|  | Linear Type II | 5.02 | 2.42 | 0.9865 | 0.9717 | 5.953 | 0.1929 | 0.1134 |
|  | Linear Type III | 4.37 | 2.61 | 0.9612 | 0.9926 | 3.899 | 0.0502 | 0.0578 |
|  | Linear Type IV | 4.2 | 2.66 | 0.9612 | 0.9949 | 3.534 | 0.0348 | 0.0481 |
|  | Nonlinear | 3.69 | 2.77 | - | 0.9975 | 3.415 | 0.0172 | 0.0338 |

**(b) Sulfate**

| Resin/pH | Langmuir Equation | K_L_ (L/mmol) | q_0_ (mmol/g) | Linear R^2^ | Nonlinear R^2^ | ARE (%) | SSE (mmol^2^/g^2^) | RMSE (mmol/g) |
| --- | --- | --- | --- | --- | --- | --- | --- | --- |
| IRA 458/4 | Linear Type I | 38.47 | 1.96 | 0.9982 | 0.9351 | 18.285 | 0.3370 | 0.1499 |
|  | Linear Type II | 20.79 | 2.83 | 0.7623 | 0.4503 | 31.188 | 2.8544 | 0.4362 |
|  | Linear Type III | 59.22 | 1.73 | 0.5664 | 0.9138 | 22.458 | 0.4474 | 0.1727 |
|  | Linear Type IV | 33.55 | 2.20 | 0.5664 | 0.8958 | 22.155 | 0.5408 | 0.1899 |
|  | Nonlinear | 47.84 | 1.93 | - | 0.9479 | 18.871 | 0.2706 | 0.1343 |
| A520E/4 | Linear Type I | 8.21 | 1.04 | 0.9917 | 0.9844 | 7.020 | 0.0153 | 0.0319 |
|  | Linear Type II | 12.32 | 0.88 | 0.9939 | 0.9492 | 5.880 | 0.0496 | 0.0575 |
|  | Linear Type III | 10.68 | 0.95 | 0.9443 | 0.9741 | 5.731 | 0.0253 | 0.0411 |
|  | Linear Type IV | 10.09 | 0.97 | 0.9443 | 0.9787 | 5.905 | 0.0208 | 0.0372 |
|  | Nonlinear | 8.05 | 1.03 | - | 0.9855 | 7.416 | 0.0141 | 0.0307 |
| IRA 67/4 | Linear Type I | 45.57 | 2.25 | 0.9974 | 0.9064 | 23.280 | 0.6765 | 0.2124 |
|  | Linear Type II | 28.13 | 2.93 | 0.6208 | 0.7067 | 30.424 | 2.1199 | 0.3759 |
|  | Linear Type III | 88.81 | 1.86 | 0.4191 | 0.8287 | 33.614 | 1.2377 | 0.2872 |
|  | Linear Type IV | 37.22 | 2.66 | 0.4191 | 0.8327 | 28.088 | 1.2090 | 0.2839 |
|  | Nonlinear | 55.74 | 2.25 | - | 0.9192 | 23.425 | 0.5842 | 0.1974 |
| IRA 96/4 | Linear Type I | 43.40 | 1.55 | 0.9990 | 0.9545 | 11.841 | 0.1293 | 0.0929 |
|  | Linear Type II | 31.79 | 1.83 | 0.8801 | 0.8119 | 16.804 | 0.5342 | 0.1887 |
|  | Linear Type III | 55.41 | 1.47 | 0.7516 | 0.9622 | 12.618 | 0.1075 | 0.0846 |
|  | Linear Type IV | 41.64 | 1.63 | 0.7516 | 0.9447 | 13.159 | 0.1569 | 0.1023 |
|  | Nonlinear | 53.32 | 1.52 | - | 0.9675 | 12.238 | 0.0923 | 0.0784 |
| IRA 458/7 | Linear Type I | 39.12 | 1.96 | 0.9982 | 0.9369 | 17.402 | 0.3248 | 0.1472 |
|  | Linear Type II | 21.99 | 2.80 | 0.8007 | 0.4290 | 30.195 | 2.9376 | 0.4425 |
|  | Linear Type III | 58.55 | 1.75 | 0.6122 | 0.9293 | 20.138 | 0.3638 | 0.1557 |
|  | Linear Type IV | 35.84 | 2.15 | 0.6122 | 0.9073 | 20.432 | 0.4769 | 0.1783 |
|  | Nonlinear | 50.27 | 1.92 | - | 0.9527 | 17.819 | 0.2435 | 0.1274 |
| A520E/7 | Linear Type I | 10.36 | 0.94 | 0.9803 | 0.9734 | 5.341 | 0.0234 | 0.0395 |
|  | Linear Type II | 13.08 | 0.86 | 0.9950 | 0.9533 | 5.272 | 0.0412 | 0.0524 |
|  | Linear Type III | 11.72 | 0.91 | 0.9455 | 0.9684 | 5.132 | 0.0278 | 0.0431 |
|  | Linear Type IV | 11.08 | 0.93 | 0.9455 | 0.9715 | 5.219 | 0.0252 | 0.0410 |
|  | Nonlinear | 9.72 | 0.96 | - | 0.9741 | 6.241 | 0.0228 | 0.0390 |
| IRA 67/7 | Linear Type I | 56.51 | 2.16 | 0.9910 | 0.8912 | 25.857 | 0.7748 | 0.2273 |
|  | Linear Type II | 30.71 | 2.83 | 0.6000 | 0.7170 | 29.806 | 2.0157 | 0.3666 |
|  | Linear Type III | 98.34 | 1.83 | 0.3858 | 0.8001 | 35.298 | 1.4237 | 0.3081 |
|  | Linear Type IV | 37.94 | 2.70 | 0.3858 | 0.7830 | 29.274 | 1.5455 | 0.3210 |
|  | Nonlinear | 59.87 | 2.23 | - | 0.8993 | 25.536 | 0.7173 | 0.2187 |
| IRA 96/7 | Linear Type I | 41.91 | 1.52 | 0.9985 | 0.9544 | 10.949 | 0.1227 | 0.0904 |
|  | Linear Type II | 33.08 | 1.75 | 0.8889 | 0.8571 | 14.536 | 0.3844 | 0.1601 |
|  | Linear Type III | 54.41 | 1.45 | 0.7667 | 0.9633 | 11.729 | 0.0988 | 0.0811 |
|  | Linear Type IV | 41.72 | 1.59 | 0.7667 | 0.9503 | 11.763 | 0.1338 | 0.0944 |
|  | Nonlinear | 52.01 | 1.50 | - | 0.9681 | 11.336 | 0.0859 | 0.0757 |
| IRA 458/10 | Linear Type I | 44.60 | 1.65 | 0.9991 | 0.9397 | 15.296 | 0.2119 | 0.1188 |
|  | Linear Type II | 24.27 | 2.27 | 0.8342 | 0.5014 | 26.432 | 1.7504 | 0.3416 |
|  | Linear Type III | 57.36 | 1.55 | 0.6419 | 0.9370 | 17.513 | 0.2211 | 0.1214 |
|  | Linear Type IV | 36.82 | 1.84 | 0.6419 | 0.8951 | 18.696 | 0.3681 | 0.1567 |
|  | Nonlinear | 52.95 | 1.65 | - | 0.9505 | 15.624 | 0.1736 | 0.1076 |
| A520E/10 | Linear Type I | 9.23 | 0.96 | 0.9864 | 0.9804 | 5.198 | 0.0172 | 0.0339 |
|  | Linear Type II | 12.07 | 0.86 | 0.9937 | 0.9567 | 5.339 | 0.0380 | 0.0503 |
|  | Linear Type III | 10.71 | 0.91 | 0.9485 | 0.9744 | 5.008 | 0.0225 | 0.0387 |
|  | Linear Type IV | 10.16 | 0.94 | 0.9485 | 0.9776 | 4.948 | 0.0196 | 0.0362 |
|  | Nonlinear | 8.73 | 0.97 | - | 0.9810 | 5.925 | 0.0166 | 0.0333 |

**Table S4.** Nonlinear Langmuir isotherm model parameters for the adsorption of (a) nitrate, (b) 3-phenylpropionic acid, and (c) sulfate on AER at different solution pH.

**(a) Nitrate**

| Resin/pH | K_L_ (L/mmol) | q_0_ (mmol/g) | R_L_ | ΔG^0^ (kJ/mol) | Nonlinear R^2^ | ARE (%) | SSE (mmol^2^/g^2^) | RMSE (mmol/g) | χ^2^ (mmol/g) |
| --- | --- | --- | --- | --- | --- | --- | --- | --- | --- |
| IRA 458/4 | 0.30 | 9.08 | 0.627 | 2.92 | 0.9881 | 2.38% | 0.093 | 0.079 | 0.038 |
| A520E/4 | 4.17 | 2.49 | 0.108 | -3.50 | 0.9968 | 3.47% | 0.018 | 0.035 | 0.024 |
| IRA 67/4 | 0.19 | 12.58 | 0.724 | 4.02 | 0.9952 | 2.53% | 0.037 | 0.050 | 0.022 |
| IRA 96/4 | 1.09 | 4.62 | 0.316 | -0.20 | 0.9984 | 1.29% | 0.013 | 0.030 | 0.006 |
| IRA 458/7 | 0.46 | 5.93 | 0.520 | 1.93 | 0.9716 | 3.71% | 0.168 | 0.106 | 0.080 |
| A520E/7 | 3.97 | 2.44 | 0.110 | -3.39 | 0.9942 | 4.05% | 0.030 | 0.045 | 0.038 |
| IRA 67/7 | 0.13 | 17.58 | 0.792 | 5.05 | 0.9959 | 2.25% | 0.034 | 0.048 | 0.017 |
| IRA 96/7 | 1.28 | 4.12 | 0.275 | -0.61 | 0.9856 | 3.67% | 0.114 | 0.087 | 0.053 |
| IRA 458/10 | 0.32 | 6.89 | 0.588 | 2.76 | 0.9971 | 1.83% | 0.016 | 0.033 | 0.009 |
| A520E/10 | 3.69 | 2.77 | 0.112 | -3.20 | 0.9975 | 3.42% | 0.017 | 0.034 | 0.031 |

**(b) 3-phenylpropionic acid (3-PPA)**

| Resin/pH | K_L_ (L/mmol) | q_0_ (mmol/g) | R_L_ | Nonlinear R^2^ | ARE (%) | SSE (mmol^2^/g^2^) | RMSE (mmol/g) | χ^2^ (mmol/g) |
| --- | --- | --- | --- | --- | --- | --- | --- | --- |
| IRA 458/4 | -0.42 | -0.28 | 11.694 | 0.9132 | 9.21 | 0.101 | 0.082 | 0.119 |
| A520E/4 | -0.32 | -0.87 | 3.180 | 0.9932 | 4.07 | 0.007 | 0.022 | 0.022 |
| IRA 67/4 | -0.52 | -0.04 | -9.908 | 0.9431 | 22.88 | 0.099 | 0.081 | 0.239 |
| IRA 96/4 | -0.38 | -0.72 | 5.362 | 0.9577 | 9.15 | 0.090 | 0.077 | 0.156 |
| IRA 458/7 | -0.30 | -1.72 | 2.922 | 0.9884 | 3.31 | 0.040 | 0.051 | 0.027 |
| A520E/7 | 0.31 | 4.24 | 0.602 | 0.9925 | 3.91 | 0.015 | 0.031 | 0.020 |
| IRA 67/7 | -0.41 | -1.13 | 8.295 | 0.9616 | 12.60 | 0.221 | 0.122 | 0.247 |
| IRA 96/7 | -0.01 | -82.12 | 1.030 | 0.8817 | 11.71 | 0.447 | 0.173 | 0.368 |
| IRA 458/10 | -0.19 | -2.76 | 1.703 | 0.9911 | 3.09 | 0.016 | 0.033 | 0.017 |
| A520E/10 | 0.60 | 2.57 | 0.436 | 0.9943 | 2.92 | 0.009 | 0.025 | 0.012 |

**(c) Sulfate**

| Resin/pH | K_L_ (L/mmol) | q_0_ (mmol/g) | R_L_ | ΔG^0^ (kJ/mol) | Nonlinear R^2^ | ARE (%) | SSE (mmol^2^/g^2^) | RMSE (mmol/g) | χ^2^ (mmol/g) |
| --- | --- | --- | --- | --- | --- | --- | --- | --- | --- |
| IRA 458/4 | 47.84 | 1.93 | 0.019 | -9.50 | 0.9479 | 18.87 | 0.271 | 0.134 | 0.416 |
| A520E/4 | 8.05 | 1.03 | 0.106 | -5.12 | 0.9855 | 7.42 | 0.014 | 0.031 | 0.039 |
| IRA 67/4 | 55.74 | 2.25 | 0.017 | -9.88 | 0.9192 | 23.43 | 0.584 | 0.197 | 0.779 |
| IRA 96/4 | 53.32 | 1.52 | 0.017 | -9.77 | 0.9675 | 12.24 | 0.092 | 0.078 | 0.165 |
| IRA 458/7 | 50.27 | 1.92 | 0.018 | -9.62 | 0.9527 | 17.82 | 0.244 | 0.127 | 0.365 |
| A520E/7 | 9.72 | 0.96 | 0.089 | -5.59 | 0.9741 | 6.24 | 0.023 | 0.039 | 0.038 |
| IRA 67/7 | 59.87 | 2.23 | 0.015 | -10.05 | 0.8993 | 25.54 | 0.717 | 0.219 | 0.873 |
| IRA 96/7 | 52.01 | 1.50 | 0.018 | -9.70 | 0.9681 | 11.34 | 0.086 | 0.076 | 0.151 |
| IRA 458/10 | 52.95 | 1.65 | 0.018 | -9.75 | 0.9505 | 15.62 | 0.174 | 0.108 | 0.283 |
| A520E/10 | 8.73 | 0.97 | 0.098 | -5.32 | 0.9810 | 5.93 | 0.017 | 0.033 | 0.032 |

**Table S5.** Nonlinear Freundlich isotherm model parameters for the adsorption of (a) nitrate, (b) 3-phenylpropionic acid, and (c) sulfate on AER at different solution pH.

**(a) Nitrate**

| Resin/pH | K_F_ (mmol^(1-1/nF^ L^1/nF^/g) | 1/n_F_ | Nonlinear R^2^ | ARE (%) | SSE (mmol^2^/g^2^) | RMSE (mmol/g) | χ^2^ (mmol/g) |
| --- | --- | --- | --- | --- | --- | --- | --- |
| IRA 458/4 | 2.09 | 0.81 | 0.9858 | 3.71 | 0.111 | 0.086 | 0.052 |
| A520E/4 | 2.02 | 0.41 | 0.9786 | 9.21 | 0.119 | 0.089 | 0.131 |
| IRA 67/4 | 2.03 | 0.86 | 0.9921 | 3.45 | 0.061 | 0.064 | 0.037 |
| IRA 96/4 | 2.36 | 0.61 | 0.9871 | 6.11 | 0.111 | 0.086 | 0.087 |
| IRA 458/7 | 1.81 | 0.73 | 0.9682 | 5.86 | 0.189 | 0.112 | 0.102 |
| A520E/7 | 1.93 | 0.40 | 0.9689 | 9.94 | 0.162 | 0.104 | 0.161 |
| IRA 67/7 | 1.98 | 0.89 | 0.9947 | 2.95 | 0.044 | 0.054 | 0.025 |
| IRA 96/7 | 2.25 | 0.56 | 0.9613 | 9.00 | 0.306 | 0.143 | 0.195 |
| IRA 458/10 | 1.66 | 0.77 | 0.9931 | 3.51 | 0.038 | 0.050 | 0.027 |
| A520E/10 | 2.16 | 0.41 | 0.9745 | 9.96 | 0.174 | 0.108 | 0.162 |

**(b) 3-phenylpropionic acid (3-PPA)**

| Resin/pH | K_F_ (mmol^(1-1/nF^ L^1/nF^/g) | 1/n_F_ | Nonlinear R^2^ | ARE (%) | SSE (mmol^2^/g^2^) | RMSE (mmol/g) | χ^2^ (mmol/g) |
| --- | --- | --- | --- | --- | --- | --- | --- |
| IRA 458/4 | 0.13 | 3.25 | 0.8949 | 13.24 | 0.122 | 0.090 | 0.202 |
| A520E/4 | 0.41 | 1.67 | 0.9778 | 7.84 | 0.023 | 0.039 | 0.072 |
| IRA 67/4 | 4.45E-05 | 16.25 | 0.9805 | 11.21 | 0.034 | 0.048 | 0.081 |
| IRA 96/4 | 0.44 | 2.07 | 0.9182 | 13.36 | 0.174 | 0.108 | 0.335 |
| IRA 458/7 | 0.74 | 1.67 | 0.9882 | 4.39 | 0.040 | 0.052 | 0.037 |
| A520E/7 | 0.97 | 0.79 | 0.9940 | 3.10 | 0.012 | 0.028 | 0.017 |
| IRA 67/7 | 0.76 | 2.28 | 0.9841 | 7.96 | 0.092 | 0.078 | 0.099 |
| IRA 96/7 | 1.27 | 0.90 | 0.9363 | 10.44 | 0.240 | 0.127 | 0.202 |
| IRA 458/10 | 0.65 | 1.36 | 0.9946 | 1.91 | 0.010 | 0.025 | 0.009 |
| A520E/10 | 0.94 | 0.67 | 0.9973 | 2.07 | 0.004 | 0.017 | 0.006 |

**(c) Sulfate**

| Resin/pH | K_F_ (mmol^(1-1/nF^ L^1/nF^/g) | 1/n_F_ | Nonlinear R^2^ | ARE (%) | SSE (mmol^2^/g^2^) | RMSE (mmol/g) | χ^2^ (mmol/g) |
| --- | --- | --- | --- | --- | --- | --- | --- |
| IRA 458/4 | 2.32 | 0.26 | 0.8674 | 29.25 | 0.688 | 0.214 | 0.825 |
| A520E/4 | 1.09 | 0.41 | 0.9914 | 6.51 | 0.008 | 0.024 | 0.029 |
| IRA 67/4 | 2.71 | 0.25 | 0.8243 | 35.34 | 1.270 | 0.291 | 1.242 |
| IRA 96/4 | 1.71 | 0.22 | 0.8779 | 21.52 | 0.347 | 0.152 | 0.506 |
| IRA 458/7 | 2.30 | 0.26 | 0.8810 | 27.18 | 0.612 | 0.202 | 0.760 |
| A520E/7 | 1.01 | 0.37 | 0.9527 | 10.12 | 0.042 | 0.053 | 0.078 |
| IRA 67/7 | 2.61 | 0.24 | 0.7756 | 37.54 | 1.599 | 0.326 | 1.442 |
| IRA 96/7 | 1.67 | 0.22 | 0.8806 | 20.56 | 0.321 | 0.146 | 0.471 |
| IRA 458/10 | 1.89 | 0.23 | 0.8587 | 25.05 | 0.496 | 0.182 | 0.674 |
| A520E/10 | 1.02 | 0.38 | 0.9633 | 9.19 | 0.032 | 0.046 | 0.064 |

**Table S6.** Nonlinear Dubinin-Radushkevich (DR) isotherm model parameters for the adsorption of (a) nitrate, (b) 3-phenylpropionic acid, and (c) sulfate on AER at different solution pH.

**(a) Nitrate**

| Resin/pH | E (kJ/mol) | q_0_ (g/g) | Nonlinear R^2^ | ARE (%) | SSE (mmol^2^/g^2^) | RMSE (mmol/g) | χ^2^ (mmol/g) |
| --- | --- | --- | --- | --- | --- | --- | --- |
| IRA 458/4 | 4.72 | 0.395 | 0.9875 | 3.27 | 0.098 | 0.081 | 0.045 |
| A520E/4 | 7.43 | 0.195 | 0.9974 | 2.89 | 0.014 | 0.031 | 0.014 |
| IRA 67/4 | 4.45 | 0.440 | 0.9974 | 1.61 | 0.020 | 0.037 | 0.010 |
| IRA 96/4 | 5.68 | 0.316 | 0.9980 | 1.55 | 0.017 | 0.034 | 0.008 |
| IRA 458/7 | 4.92 | 0.317 | 0.9717 | 3.32 | 0.168 | 0.106 | 0.079 |
| A520E/7 | 7.41 | 0.188 | 0.9924 | 4.46 | 0.040 | 0.051 | 0.037 |
| IRA 67/7 | 4.32 | 0.466 | 0.9960 | 1.66 | 0.033 | 0.047 | 0.014 |
| IRA 96/7 | 5.85 | 0.291 | 0.9824 | 4.38 | 0.139 | 0.096 | 0.067 |
| IRA 458/10 | 4.67 | 0.324 | 0.9974 | 1.70 | 0.014 | 0.031 | 0.009 |
| A520E/10 | 7.29 | 0.214 | 0.9962 | 3.10 | 0.026 | 0.042 | 0.021 |

**(b) 3-phenylpropionic acid (3-PPA)**

| Resin/pH | E (kJ/mol) | q_0_ (g/g) | Nonlinear R^2^ | ARE (%) | SSE (mmol^2^/g^2^) | RMSE (mmol/g) | χ^2^ (mmol/g) |
| --- | --- | --- | --- | --- | --- | --- | --- |
| IRA 458/4 | 1.57 | 2.550 | 0.8808 | 14.85 | 0.138 | 0.096 | 0.265 |
| A520E/4 | 2.39 | 0.561 | 0.9521 | 11.76 | 0.050 | 0.058 | 0.178 |
| IRA 67/4 | 0.67 | 7.99E+05 | 0.9811 | 10.73 | 0.033 | 0.047 | 0.078 |
| IRA 96/4 | 2.09 | 1.140 | 0.8834 | 16.38 | 0.248 | 0.129 | 0.562 |
| IRA 458/7 | 2.37 | 1.036 | 0.9780 | 7.17 | 0.075 | 0.071 | 0.106 |
| A520E/7 | 3.90 | 0.347 | 0.9839 | 5.49 | 0.032 | 0.046 | 0.052 |
| IRA 67/7 | 2.02 | 2.442 | 0.9904 | 5.61 | 0.055 | 0.061 | 0.056 |
| IRA 96/7 | 3.40 | 0.586 | 0.9669 | 6.66 | 0.125 | 0.091 | 0.093 |
| IRA 458/10 | 2.61 | 0.620 | 0.9930 | 3.48 | 0.013 | 0.029 | 0.017 |
| A520E/10 | 4.31 | 0.289 | 0.9877 | 4.73 | 0.019 | 0.036 | 0.031 |

**(c) Sulfate**

| Resin/pH | E (kJ/mol) | q_0_ (g/g) | Nonlinear R^2^ | ARE (%) | SSE (mmol^2^/g^2^) | RMSE (mmol/g) | χ^2^ (mmol/g) |
| --- | --- | --- | --- | --- | --- | --- | --- |
| IRA 458/4 | 10.44 | 0.241 | 0.9081 | 24.28 | 0.477 | 0.178 | 0.645 |
| A520E/4 | 7.70 | 0.129 | 0.9983 | 1.84 | 0.002 | 0.011 | 0.003 |
| IRA 67/4 | 10.71 | 0.280 | 0.8730 | 30.49 | 0.918 | 0.247 | 1.010 |
| IRA 96/4 | 11.23 | 0.179 | 0.9151 | 18.12 | 0.241 | 0.127 | 0.372 |
| IRA 458/7 | 10.55 | 0.238 | 0.9170 | 22.64 | 0.427 | 0.169 | 0.593 |
| A520E/7 | 8.05 | 0.119 | 0.9751 | 4.64 | 0.022 | 0.038 | 0.030 |
| IRA 67/7 | 11.00 | 0.272 | 0.8345 | 32.77 | 1.179 | 0.280 | 1.164 |
| IRA 96/7 | 11.25 | 0.175 | 0.9182 | 17.46 | 0.220 | 0.121 | 0.341 |
| IRA 458/10 | 10.99 | 0.196 | 0.8969 | 21.69 | 0.362 | 0.155 | 0.530 |
| A520E/10 | 7.87 | 0.121 | 0.9833 | 3.65 | 0.015 | 0.031 | 0.020 |

**Table S7.** Nonlinear Dubinin-Astakhov (DA) isotherm model parameters for the adsorption of (a) nitrate, (b) 3-phenylpropionic acid, and (c) sulfate on AER at different solution pH.

**(a) Nitrate**

| Resin/pH | E (kJ/mol) | n_D_ | Nonlinear R^2^ | ARE (%) | SSE (mmol^2^/g^2^) | RMSE (mmol/g) | χ^2^ (mmol/g) |
| --- | --- | --- | --- | --- | --- | --- | --- |
| IRA 458/4 | 5.73 | 2.97 | 0.9801 | 5.92 | 0.156 | 0.102 | 0.100 |
| A520E/4 | 8.39 | 2.98 | 0.9967 | 3.15 | 0.019 | 0.035 | 0.019 |
| IRA 67/4 | 5.40 | 2.97 | 0.9943 | 3.24 | 0.044 | 0.054 | 0.033 |
| IRA 96/4 | 6.89 | 3.43 | 0.9900 | 5.39 | 0.086 | 0.076 | 0.075 |
| IRA 458/7 | 5.46 | 2.46 | 0.9701 | 4.20 | 0.177 | 0.109 | 0.086 |
| A520E/7 | 8.23 | 2.86 | 0.9947 | 3.89 | 0.027 | 0.043 | 0.030 |
| IRA 67/7 | 5.34 | 3.11 | 0.9888 | 4.20 | 0.092 | 0.079 | 0.055 |
| IRA 96/7 | 6.86 | 3.25 | 0.9873 | 3.70 | 0.100 | 0.082 | 0.048 |
| IRA 458/10 | 5.24 | 2.50 | 0.9956 | 2.69 | 0.024 | 0.040 | 0.019 |
| A520E/10 | 8.50 | 3.56 | 0.9866 | 7.06% | 0.092 | 0.078 | 0.111 |

**(b) 3-phenylpropionic acid (3-PPA)**

| Resin/pH | E (kJ/mol) | n_D_ | Nonlinear R^2^ | ARE (%) | SSE (mmol^2^/g^2^) | RMSE (mmol/g) | χ^2^ (mmol/g) |
| --- | --- | --- | --- | --- | --- | --- | --- |
| IRA 458/4 | 2.37 | 3.36 | 0.8622 | 16.38 | 0.160 | 0.103 | 0.311 |
| A520E/4 | 2.83 | 2.48 | 0.9387 | 13.12 | 0.064 | 0.066 | 0.187 |
| IRA 67/4 | 2.58 | 15.91 | 0.9775 | 8.15 | 0.039 | 0.051 | 0.081 |
| IRA 96/4 | 2.81 | 2.92 | 0.8507 | 18.41 | 0.318 | 0.146 | 0.563 |
| IRA 458/7 | 2.92 | 2.72 | 0.9678 | 8.59 | 0.110 | 0.086 | 0.151 |
| A520E/7 | 3.79 | 1.86 | 0.9859 | 5.04 | 0.028 | 0.043 | 0.039 |
| IRA 67/7 | 3.06 | 4.12 | 0.9912 | 4.01 | 0.051 | 0.058 | 0.046 |
| IRA 96/7 | 3.70 | 2.46 | 0.9740 | 4.94 | 0.098 | 0.081 | 0.063 |
| IRA 458/10 | 2.61 | 2.00 | 0.9929 | 3.44 | 0.013 | 0.029 | 0.017 |
| A520E/10 | 3.72 | 1.54 | 0.9942 | 2.59 | 0.009 | 0.025 | 0.010 |

**(c) Sulfate**

| Resin/pH | E (kJ/mol) | n_D_ | Nonlinear R^2^ | ARE (%) | SSE (mmol^2^/g^2^) | RMSE (mmol/g) | χ^2^ (mmol/g) |
| --- | --- | --- | --- | --- | --- | --- | --- |
| IRA 458/4 | 11.49 | 3.78 | 0.9402 | 20.08 | 0.310 | 0.144 | 0.453 |
| A520E/4 | 8.11 | 2.26 | 0.9972 | 1.85 | 0.003 | 0.013 | 0.004 |
| IRA 67/4 | 11.95 | 4.86 | 0.9201 | 23.66 | 0.578 | 0.196 | 0.757 |
| IRA 96/4 | 11.69 | 3.47 | 0.9455 | 15.26 | 0.155 | 0.102 | 0.230 |
| IRA 458/7 | 11.54 | 3.61 | 0.9436 | 19.07 | 0.290 | 0.139 | 0.418 |
| A520E/7 | 8.10 | 2.06 | 0.9756 | 4.08 | 0.022 | 0.038 | 0.027 |
| IRA 67/7 | 12.05 | 5.38 | 0.8995 | 24.53 | 0.716 | 0.218 | 0.840 |
| IRA 96/7 | 11.61 | 3.09 | 0.9423 | 15.22 | 0.155 | 0.102 | 0.235 |
| IRA 458/10 | 10.94 | 2.12 | 0.8985 | 21.19 | 0.356 | 0.154 | 0.515 |
| A520E/10 | 8.00 | 2.11 | 0.9840 | 3.02% | 0.014 | 0.031 | 0.018 |

**Table S8.** Nonlinear Redlich-Peterson (RP) isotherm model parameters for the adsorption of (a) nitrate, (b) 3-phenylpropionic acid, and (c) sulfate on AER at different solution pH.

**(a) Nitrate**

| Resin/pH | $K_{RP}$ (L/g) | $b_{RP}$ (L/mmol)^α^ | α | Nonlinear R^2^ | ARE (%) | SSE (mmol^2^/g^2^) | RMSE (mmol/g) | χ^2^ (mmol/g) |
| --- | --- | --- | --- | --- | --- | --- | --- | --- |
| IRA 458/4 | 2.76 | 0.30 | 1.00 | 0.9881 | 2.38 | 0.093 | 0.079 | 0.038 |
| A520E/4 | 13.04 | 5.49 | 0.88 | 0.9991 | 1.16 | 0.005 | 0.018 | 0.003 |
| IRA 67/4 | 2.45 | 0.19 | 1.00 | 0.9952 | 2.53 | 0.037 | 0.050 | 0.022 |
| IRA 96/4 | 5.02 | 1.09 | 1.00 | 0.9984 | 1.29 | 0.013 | 0.030 | 0.006 |
| IRA 458/7 | 2.70 | 0.46 | 1.00 | 0.9716 | 3.71 | 0.168 | 0.106 | 0.080 |
| A520E/7 | 11.13 | 4.73 | 0.92 | 0.9952 | 3.77 | 0.025 | 0.041 | 0.025 |
| IRA 67/7 | 2.25 | 0.13 | 1.00 | 0.9959 | 2.25 | 0.034 | 0.048 | 0.017 |
| IRA 96/7 | 5.28 | 1.28 | 1.00 | 0.9856 | 3.67 | 0.114 | 0.087 | 0.053 |
| IRA 458/10 | 2.24 | 0.32 | 1.00 | 0.9971 | 1.83 | 0.016 | 0.033 | 0.009 |
| A520E/10 | 11.78 | 4.41 | 0.92 | 0.9984 | 2.05 | 0.011 | 0.027 | 0.014 |

**(b) 3-phenylpropionic acid (3-PPA)**

| Resin/pH | $K_{RP}$ (L/g) | $b_{RP}$ (L/mmol)^α^ | α | Nonlinear R^2^ | ARE (%) | SSE (mmol^2^/g^2^) | RMSE (mmol/g) | χ^2^ (mmol/g) |
| --- | --- | --- | --- | --- | --- | --- | --- | --- |
| IRA 458/4 | 0.12 | -0.42 | 1.00 | 0.9132 | 9.21 | 0.101 | 0.082 | 0.119 |
| A520E/4 | 0.28 | -0.32 | 1.00 | 0.9932 | 4.07 | 0.007 | 0.022 | 0.022 |
| IRA 67/4 | 0.002 | -0.95 | 0.08 | 0.9463 | 22.15 | 0.093 | 0.079 | 0.226 |
| IRA 96/4 | 0.28 | -0.38 | 1.00 | 0.9577 | 9.15 | 0.090 | 0.077 | 0.156 |
| IRA 458/7 | 0.17 | -0.78 | 0.16 | 0.9896 | 3.10 | 0.036 | 0.049 | 0.025 |
| A520E/7 | 3.75 | 2.84 | 0.29 | 0.9940 | 3.17 | 0.012 | 0.028 | 0.017 |
| IRA 67/7 | 0.002 | -1.00 | 0.002 | 0.9727 | 10.52 | 0.158 | 0.102 | 0.178 |
| IRA 96/7 | 1.46 | 0.13 | 1.00 | 0.9431 | 10.27 | 0.215 | 0.120 | 0.190 |
| IRA 458/10 | 0.002 | -1.00 | 0.001 | 0.9939 | 2.20 | 0.011 | 0.027 | 0.010 |
| A520E/10 | 3.56 | 2.75 | 0.47 | 0.9977 | 1.57 | 0.004 | 0.016 | 0.004 |

**(c) Sulfate**

| Resin/pH | $K_{RP}$ (L/g) | $b_{RP}$ (L/mmol)^α^ | α | Nonlinear R^2^ | ARE (%) | SSE (mmol^2^/g^2^) | RMSE (mmol/g) | χ^2^ (mmol/g) |
| --- | --- | --- | --- | --- | --- | --- | --- | --- |
| IRA 458/4 | 92.46 | 47.84 | 1.00 | 0.9479 | 18.87 | 0.271 | 0.134 | 0.416 |
| A520E/4 | 17.13 | 15.71 | 0.74 | 0.9992 | 1.60 | 0.001 | 0.007 | 0.002 |
| IRA 67/4 | 125.29 | 55.74 | 1.00 | 0.9192 | 23.43 | 0.584 | 0.197 | 0.779 |
| IRA 96/4 | 81.28 | 53.32 | 1.00 | 0.9675 | 12.24 | 0.092 | 0.078 | 0.165 |
| IRA 458/7 | 96.46 | 50.27 | 1.00 | 0.9527 | 17.82 | 0.243 | 0.127 | 0.365 |
| A520E/7 | 12.20 | 12.36 | 0.88 | 0.9774 | 3.27 | 0.020 | 0.036 | 0.024 |
| IRA 67/7 | 133.44 | 59.87 | 1.00 | 0.8993 | 25.54 | 0.717 | 0.219 | 0.873 |
| IRA 96/7 | 77.83 | 52.01 | 1.00 | 0.9681 | 11.34 | 0.086 | 0.076 | 0.151 |
| IRA 458/10 | 87.17 | 52.95 | 1.00 | 0.9505 | 15.62 | 0.174 | 0.108 | 0.283 |
| A520E/10 | 11.45 | 11.40 | 0.87 | 0.9847 | 2.84 | 0.013 | 0.030 | 0.017 |

**Fig. S1.** Perfluoroalkyl acids (PFAAs) removal by (a, c) polyacrylic (PA), and (b, d) polystyrene (PS) AER at pH 10 in the presence of sodium bicarbonate ($C_{0}\approx2.14 meq/L)$. Initial concentration of each PFAS was $C_{0}=80 \mu g/L$ ($\sum\mathrm{PFAS}=480 \mu g/L)$.

**Fig. S2.** Equilibrium adsorption isotherms of nitrate onto (a, c) polyacrylic and (b, d) polystyrene anion exchange resins at pH 4.

**Fig. S3.** Equilibrium adsorption isotherms of sulfate onto (a, c) polyacrylic and (b, d) polystyrene anion exchange resins at pH 4.

**Fig. S4.** Equilibrium adsorption isotherms of (a, b) nitrate, (c, d) 3-phenylpropionic acid (3-PPA), and (e, f) sulfate onto polyacrylic (left panels) and polystyrene (right panels) strong-base anion exchange resins at pH 10.

**Fig. S5.** Equilibrium exchange plots of nitrate at (a) pH 4, (b) pH 7 and (c) pH 10 with chloride-form anion exchange resins in single-solute systems.

**Fig. S6.** Equilibrium exchange plots of 3-phenylpropionic acid at (a) pH 4, (b) pH 7, and (c) pH 10 with chloride-form anion exchange resins in single-solute systems.

**Fig. S7.** Equilibrium exchange plots of sulfate at (a) pH 4, (b) pH 7 and (c) pH 10 with chloride-form anion exchange resins in single-solute systems.

**Fig. S8.** Equivalent chloride release and corresponding uptake of (a, b) nitrate, (c, d) 3-phenylpropionic acid (3-PPA), and (e, f) sulfate onto strong-base (left panels) and weak-base (right panels) anion exchange resins at pH 4.

**Fig. S9.** Equivalent chloride release and corresponding uptake of (a, b) nitrate, (c, d) 3-phenylpropionic acid (3-PPA), and (e, f) sulfate onto strong-base (left panels) and weak-base (right panels) anion exchange resins at pH 7.

**Fig. S10.** Equivalent chloride release and corresponding uptake of (a, b) nitrate, (c, d) 3-phenylpropionic acid (3-PPA), and (e, f) sulfate onto strong-base anion exchange resins at pH 10.

**References**

Ahmed, M.J. and Dhedan, S.K. 2012. Equilibrium isotherms and kinetics modeling of methylene blue adsorption on agricultural wastes-based activated carbons. Fluid Phase Equilibria 317, 9-14.

Allen, S.J., Gan, Q., Matthews, R. and Johnson, P.A. 2003. Comparison of optimised isotherm models for basic dye adsorption by kudzu. Bioresource Technology 88(2), 143-152.

Bolster, C.H. and Hornberger, G.M. 2007. On the Use of Linearized Langmuir Equations. Soil Science Society of America Journal 71(6), 1796-1806.

Chan, L.S., Cheung, W.H., Allen, S.J. and McKay, G. 2012. Error Analysis of Adsorption Isotherm Models for Acid Dyes onto Bamboo Derived Activated Carbon. Chinese Journal of Chemical Engineering 20(3), 535-542.

Chen, R., Yang, Q., Zhong, Y., Li, X., Liu, Y., Li, X.-M., Du, W.-X. and Zeng, G.-M. 2014. Sorption of trace levels of bromate by macroporous strong base anion exchange resin: Influencing factors, equilibrium isotherms and thermodynamic studies. Desalination 344, 306-312.

Dron, J. and Dodi, A. 2011. Comparison of adsorption equilibrium models for the study of CL−, NO3− and SO42− removal from aqueous solutions by an anion exchange resin. Journal of Hazardous Materials 190(1), 300-307.

Dubinin, M. 1947 The equation of the characteristic curve of activated charcoal, pp. 327-329.

El-Khaiary, M.I. 2008. Least-squares regression of adsorption equilibrium data: Comparing the options. Journal of Hazardous Materials 158(1), 73-87.

Foo, K.Y. and Hameed, B.H. 2010. Insights into the modeling of adsorption isotherm systems. Chemical Engineering Journal 156(1), 2-10.

Freundlich, H. 1906. Over the adsorption in solution. J. Phys. chem 57(385471), 1100-1107.

Fu, L., Wang, J., Lu, H., Su, Y. and Ren, A. 2008. Comment on “The removal of phenolic compounds from aqueous solutions by organophilic bentonite”. Journal of Hazardous Materials 151(2), 851-854.

Helfferich, F.G. (1995) Ion exchange, Courier Corporation.

Howe, K.J., Hand, D.W., Crittenden, J.C., Trussell, R.R. and Tchobanoglous, G. (2012) Principles of water treatment, John Wiley & Sons.

Hu, Y., Foster, J. and Boyer, T.H. 2016. Selectivity of bicarbonate-form anion exchange for drinking water contaminants: Influence of resin properties. Separation and Purification Technology 163, 128-139.

Inglezakis, V.J. 2007. Solubility-normalized Dubinin–Astakhov adsorption isotherm for ion-exchange systems. Microporous and Mesoporous Materials 103(1), 72-81.

Jossens, L., Prausnitz, J.M., Fritz, W., Schlünder, E.U. and Myers, A.L. 1978. Thermodynamics of multi-solute adsorption from dilute aqueous solutions. Chemical Engineering Science 33(8), 1097-1106.

Kinniburgh, D.G. 1986. General purpose adsorption isotherms. Environmental science & technology 20(9), 895-904.

Kumar, K.V. and Sivanesan, S. 2005. Comparison of linear and non-linear method in estimating the sorption isotherm parameters for safranin onto activated carbon. Journal of Hazardous Materials 123(1), 288-292.

Landry, K.A., Sun, P., Huang, C.-H. and Boyer, T.H. 2015. Ion-exchange selectivity of diclofenac, ibuprofen, ketoprofen, and naproxen in ureolyzed human urine. Water Research 68, 510-521.

Langmuir, I. 1916. The constitution and fundamental properties of solids and liquids. Part I. Solids. Journal of the American chemical society 38(11), 2221-2295.

Milmile, S.N., Pande, J.V., Karmakar, S., Bansiwal, A., Chakrabarti, T. and Biniwale, R.B. 2011. Equilibrium isotherm and kinetic modeling of the adsorption of nitrates by anion exchange Indion NSSR resin. Desalination 276(1), 38-44.

Namasivayam, C. and Ranganathan, K. 1995. Removal of Cd(II) from wastewater by adsorption on “waste” Fe(III)Cr(III) hydroxide. Water Research 29(7), 1737-1744.

Onyango, M.S., Kojima, Y., Aoyi, O., Bernardo, E.C. and Matsuda, H. 2004. Adsorption equilibrium modeling and solution chemistry dependence of fluoride removal from water by trivalent-cation-exchanged zeolite F-9. Journal of Colloid and Interface Science 279(2), 341-350.

Özcan, A., Özcan, A.S., Tunali, S., Akar, T. and Kiran, I. 2005. Determination of the equilibrium, kinetic and thermodynamic parameters of adsorption of copper(II) ions onto seeds of Capsicum annuum. Journal of Hazardous Materials 124(1), 200-208.

Park, M., Daniels, K.D., Wu, S., Ziska, A.D. and Snyder, S.A. 2020. Magnetic ion-exchange (MIEX) resin for perfluorinated alkylsubstance (PFAS) removal in groundwater: Roles of atomic charges for adsorption. Water Research 181, 115897.

Radke, C. and Prausnitz, J. 1972. Adsorption of organic solutes from dilute aqueous solution of activated carbon. Industrial & Engineering Chemistry Fundamentals 11(4), 445-451.

Tran, H.N., You, S.-J., Hosseini-Bandegharaei, A. and Chao, H.-P. 2017. Mistakes and inconsistencies regarding adsorption of contaminants from aqueous solutions: A critical review. Water Research 120, 88-116.

Zeng, C., Atkinson, A., Sharma, N., Ashani, H., Hjelmstad, A., Venkatesh, K. and Westerhoff, P. 2020. Removing per- and polyfluoroalkyl substances from groundwaters using activated carbon and ion exchange resin packed columns. AWWA Water Science 2(1), e1172.
